# Differential regulation of PKD isoforms in oxidative stress conditions through phosphorylation of a conserved Tyr in the P+1 loop

**DOI:** 10.1038/s41598-017-00800-w

**Published:** 2017-04-20

**Authors:** Mathias Cobbaut, Rita Derua, Heike Döppler, Hua Jane Lou, Sandy Vandoninck, Peter Storz, Benjamin E. Turk, Thomas Seufferlein, Etienne Waelkens, Veerle Janssens, Johan Van Lint

**Affiliations:** 1 Department of Cellular and Molecular Medicine, Faculty of Medicine, KU Leuven, Leuven Belgium; 2 Leuven Cancer Institute (LKI), KU Leuven, Leuven Belgium; 3 Department of Cancer Biology, Mayo Clinic, Jacksonville, FL USA; 4 Department of Pharmacology, Yale School of Medicine, New Haven, Connecticut USA; 5 Department of Internal Medicine I, University of Ulm, Ulm, Germany

## Abstract

Protein kinases are essential molecules in life and their crucial function requires tight regulation. Many kinases are regulated via phosphorylation within their activation loop. This loop is embedded in the activation segment, which additionally contains the Mg^2+^ binding loop and a P + 1 loop that is important in substrate binding. In this report, we identify Abl-mediated phosphorylation of a highly conserved Tyr residue in the P + 1 loop of protein kinase D2 (PKD2) during oxidative stress. Remarkably, we observed that the three human PKD isoforms display very different degrees of P + 1 loop Tyr phosphorylation and we identify one of the molecular determinants for this divergence. This is paralleled by a different activation mechanism of PKD1 and PKD2 during oxidative stress. Tyr phosphorylation in the P + 1 loop of PKD2 increases turnover for Syntide-2, while substrate specificity and the role of PKD2 in NF-κB signaling remain unaffected. Importantly, Tyr to Phe substitution renders the kinase inactive, jeopardizing its use as a non-phosphorylatable mutant. Since large-scale proteomics studies identified P + 1 loop Tyr phosphorylation in more than 70 Ser/Thr kinases in multiple conditions, our results do not only demonstrate differential regulation/function of PKD isoforms under oxidative stress, but also have implications for kinase regulation in general.

## Introduction

Protein kinases are essential as receivers, transmitters and executors of a wide variety of cellular stimuli. Their activation results in a plethora of biological responses such as cellular movement, proliferation, and differentiation^1^. Proper control of these processes requires tight regulation, and deregulation of kinase activity causes a variety of diseases^2^.

The activity of many kinases is regulated via the conformation of their activation segment, which is defined as the region between the conserved DFG and APE motifs^3, 4^. The activation segment can be subdivided into the Mg^2+^ binding loop, the activation loop and the P + 1 loop^5^. In many kinases, the activation loop is phosphorylated, inducing a conformational change of the activation segment, thereby establishing the active conformation^3, 4^. The P + 1 loop was originally named for its involvement in contacts with the P + 1 amino acid residue in Protein Kinase A (PKA) substrates (i.e. the first residue C-terminal of the phospho-acceptor), but actually makes extended contacts with the substrate. Remarkably, while phosphorylation events in the activation loop are well-documented, kinases can also be phosphorylated in their P + 1 loops^6–42^. This phenomenon has become increasingly clear as a consequence of large scale proteomics studies that give unprecedented insight in post-translational modifications (PTMs) in a variety of proteins. However, in most cases the functional consequences of these P + 1 loop phosphorylation events remain undefined.

The protein kinase D (PKD) family belongs to the CAMK group of the kinome and consists of three highly homologous members (PKD1, 2, and 3) in humans. They have a modular structure, consisting of two diacylglycerol (DAG) binding C1 domains that connect via an acidic stretch to a PH domain, followed by the kinase catalytic domain^43^. The activity of PKD is regulated through auto-inhibition by the C1 and PH domains. Classically, PKDs are DAG responders that in many cases signal downstream of PKC pathways^44^. They bind at DAG-containing membranes through their C1 domains, where they co-localize with PKC isoforms. PKC subsequently phosphorylates the activation loop Ser-738/742 residues (hPKD1 numbering), resulting in alleviation of auto-inhibition of the PH domain and activation of PKD^45^.

PKDs are also responsive to oxidative stress conditions. Here, PKD1 activation is dependent on the hierarchical phosphorylation of several tyrosine residues. First, Tyr-463 in the PH domain is phosphorylated by Abl^46^. Tyr-463 phosphorylation induces a conformation permissive for subsequent Src-mediated phosphorylation of Tyr-95 in the N-terminus of PKD1^46, 47^. Phospho-Tyr-95 serves as a docking site for the C2 domain of PKCδ, which phosphorylates PKD1 at its activation loop Ser-738/742 residues, an event that has been shown to be essential for PKD1 activation under oxidative stress^47–49^. One of the downstream effectors of stress activated PKD1 is the NF-κB pathway. PKD1 activates the transcriptional activity of NF-κB target genes via the IKK complex; however a direct target of PKD1 in this pathway remains elusive^48, 50^. Activation of NF-κB via mitochondrial ROS results in expression of MnSOD, thereby detoxifying the cell from damaging ROS^51^. On the other hand, PKD activated under oxidative stress conditions also increases JNK activity downstream of DAPK in a PKC- and pSer-738/742 independent manner, promoting cell death^52, 53^.

Studies on the activation mechanisms of PKD enzymes by tyrosine phosphorylation have been largely limited to PKD1. However, under oxidative stress conditions, PKD2 is phosphorylated at Tyr-87^47^. Furthermore, PKD2 is phosphorylated at tyrosine residues by the BCR-Abl fusion protein in BCR-Abl^+^ CML cell lines^54^. BCR-Abl phosphorylates PKD2 at Tyr-438 in its PH-domain (the site analogous to Tyr-463 that is phosphorylated in oxidative stress conditions in PKD1).

In this study, we reveal phosphorylation of a key tyrosine residue in the P + 1 loop of PKD2 under oxidative stress conditions. Despite its very high conservation, we observe remarkable isoform-specific phosphorylation, revealing unprecedented insight into differential regulation of the PKD family that impacts on their activity towards substrates.

## Results

### PKD2 is phosphorylated at Tyr-717 in the activation segment under oxidative stress conditions

**Figure 1:**
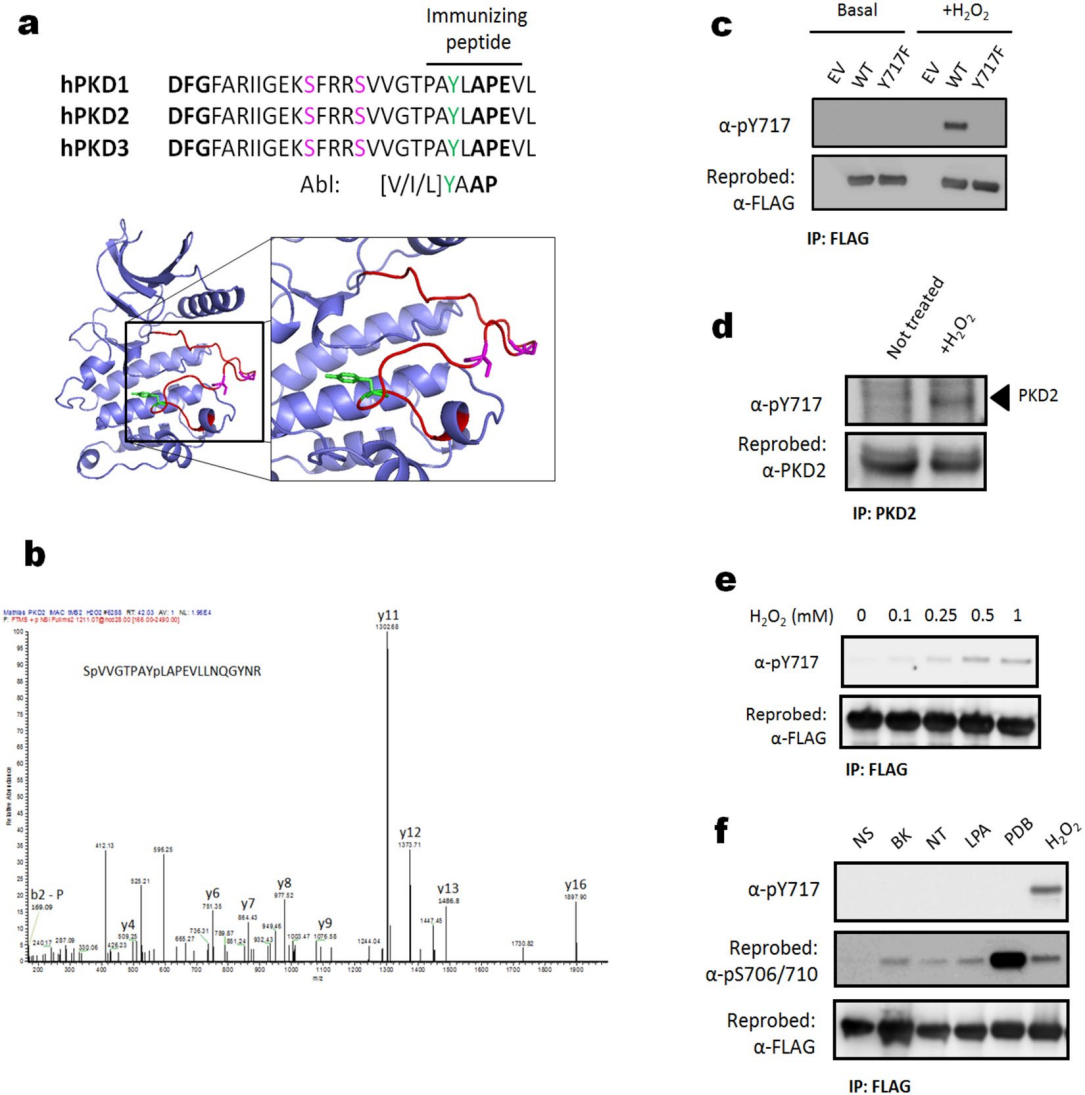
Identification of PKD2 Tyr-717 phosphorylation during oxidative stress. (**a**) Upper panel: alignment of the PKD1/2/3 activation segment. Activation loop serine residues are denoted in purple, the identified pTyr-residue in green. The borders of the activation segment are indicated in bold. Lower panel: position of the activation loop Ser residues and Tyr residue in a PKD2 homology model (generated by the Phyre2 server). (**b**) Mass spectrometry based identification of the p-Tyr717 containing peptide following oxidative stress. (**c**) Tyr-717 phosphorylation after oxidative stress is detected by a PSSA. HEK293 cells were transfected with wild type (WT) PKD2 or an Y717F mutant. 48 h after transfection, cells were stimulated with H_2_O_2_ (10 mM, 10 min) and PKD2 was precipitated from the cells using FLAG antibody. Phosphorylation of Tyr-717 was assessed using the home-made PSSA. Western blots were cropped for clarity; uncropped images can be found in Supplementary Fig. S4. (**d**) Phosphorylation of endogenous PKD2 following oxidative stress. HEK293 cells were stimulated with H_2_O_2_ (10 mM, 10 min) and PKD2 was precipitated from the cells using a PKD2 antibody. Phosphorylation of Tyr-717 was assessed using the home-made PSSA. N.S.: non-stimulated cells. Western blots were cropped for clarity; uncropped images can be found in Supplementary Fig. S5. (**e**) Sub-millimolar doses of H_2_O_2_ elicit Tyr-717 phosphorylation. HEK293 cells were transfected with wild type (WT) PKD2. 48 h after transfection, cells were stimulated with H_2_O_2_ at the indicated concentrations for 10 min and PKD2 was precipitated from the cells using FLAG antibody. Phosphorylation of Tyr-717 was assessed using the home-made PSSA. Western blots were cropped for clarity; uncropped images can be found in Supplementary Fig. S6. (**f**) Tyr-717 phosphorylation is not observed in classical PKD activation by GPCRs or phorbol-12,13-dibutyrate (PDB). HEK293 cells were transfected with wild type (WT) PKD2. 48 h after transfection, cells were serum starved for 6 h prior to stimulation with bradykinin (BK; 1 µM, 5 min), neurotensin (NT; 1 µM, 10 min), Lysophosphatidic acid (LPA; 10 µM, 5 min), PDB (500 nM, 15 min) or H_2_O_2_ (10 mM, 10 min). FLAG immunoprecipitates were subjected to western blot and probed with the indicated antibodies. Western blots were cropped for clarity; uncropped images can be found in Supplementary Fig. S7.

PKD2 undergoes many post-translational modifications (25 sites are reported as phosphorylated in the Phosphosite database (www.phosphosite.org), selecting those found in at least 5 independent studies, see Supplementary Table S1), the effect of which in the majority of cases is not known. Interestingly, we noticed that phosphorylation of Tyr-717 in the activation segment P + 1 loop, just before the highly conserved APE motif, was detected in as many as 39 proteomic data sets (Fig. 1a). However, the role of this phosphorylation event has never been addressed. Because the PKD family of kinases is regulated by tyrosine phosphorylation during oxidative stress, we investigated whether Tyr-717 could also be phosphorylated under these conditions. Mass spectrometric analysis of PKD2 purified from HEK293 cells stimulated with H_2_O_2_ indicated that indeed, Tyr-717 is a target site on PKD2 under oxidative stress conditions (Fig. 1b). To study this phenomenon in further detail, we generated a phosphorylation site-specific antibody (PSSA) directed against this site. As shown in Fig. 1c, PKD2 stimulated by H_2_O_2_ was recognized by the PSSA, while no Tyr-717 phosphorylation could be detected under basal conditions or in a stimulated enzyme where Tyr-717 is substituted by Phe. Furthermore, we could also detect phosphorylation on Tyr-717 in endogenous PKD2 after oxidative stress (Fig. 1d). Tyr-717 phosphorylation could also be detected with sub-millimolar doses of H_2_O_2_ (Fig. 1e), but no phosphorylation was seen during classical activation of PKD2 with GPCR agonists or phorbol-12,13-dibutyrate (PDB) (Fig. 1f).

### Abl acts as an upstream kinase for Tyr-717 under oxidative stress conditions

**Figure 2:**
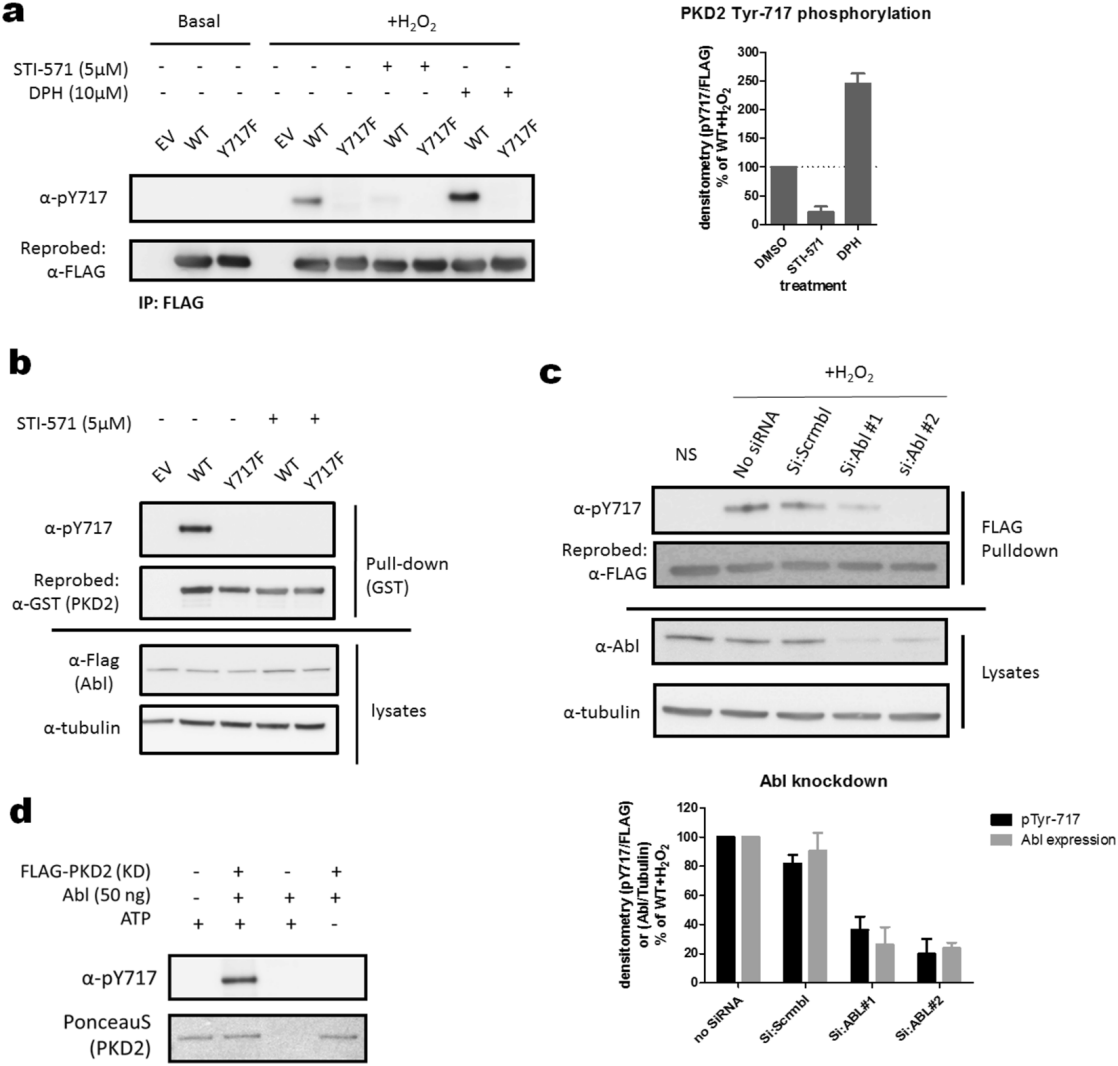
Abl is an upstream kinase for Tyr717 in PKD2. (**a**) Effect of the Abl inhibitor STI-571 and activator DPH on the phosphorylation of Tyr-717 following oxidative stress. HEK293 cells were transfected with wild type (WT) PKD2 or an Y717F mutant. 48 h after transfection, cells were treated with the indicated compounds and stimulated with H_2_O_2_ (10 mM, 10 min) and PKD2 was precipitated from the cells using FLAG antibody. Phosphorylation of Tyr-717 was assessed using the home-made PSSA. Quantification of three independent experiments is shown. Western blots were cropped for clarity; uncropped images can be found in Supplementary Fig. S8 (**b**) Co-transfection of c-Abl with PKD2 results in Tyr-717 phosphorylation without additional stimulus. HEK293 cells were transfected with GST-tagged wild type (WT) PKD2 or an Y717F mutant and FLAG-tagged c-Abl. 48 h after transfection, PKD2 was precipitated from the cells using Glutathione-sepharose beads. Phosphorylation of Tyr-717 was assessed using the home-made PSSA. Western blots were cropped for clarity; uncropped images can be found in Supplementary Fig. S9 (**c**) siRNA mediated downregulation of Abl expression results in decreased Tyr-717 phosphorylation of PKD2. Cells were transfected with siRNA targeted against c-Abl and subsequently transfected with FLAG-PKD2. PKD2 was precipitated from the cells using FLAG antibody and phosphorylation on Tyr-717 was followed with the PSSA. Quantification of three independent experiments is shown. Western blots were cropped for clarity; uncropped images can be found in Supplementary Fig. S10 (**d**) *in vitro* kinase assay showing direct phosphorylation of PKD2 by Abl. PKD2 (kinase dead, K580A mutant) was incubated with or without 50 ng Abl and incubated for 30 min at 30 °C. Phosphorylation of Tyr-717 was assessed using the home-made PSSA. Western blots were cropped for clarity; uncropped images can be found in Supplementary Fig. S11.

PKD1 is Tyr phosphorylated by both Src and Abl kinases during oxidative stress. For Tyr-717 in PKD2, Abl was an intriguing candidate, since the motif surrounding Tyr-717 corresponds to the preferred Abl recognition sequence (Fig. 1a)^55^. Furthermore, Abl has already been identified as an upstream kinase for tyrosine phosphorylation of Tyr-438 in PKD2^54^. To test whether Abl could act as an upstream kinase for Tyr-717 in oxidative stress, we pre-incubated cells with a pharmacological inhibitor (STI-571) or activator (DPH) of Abl, and then stimulated them with H_2_O_2_. Phosphorylation of Tyr-717 was markedly decreased in presence of STI-571, while incubation with DPH, resulting in stronger Abl activation, enhanced Tyr-717 phosphorylation even further (Fig. 2a). Furthermore, co-transfection of PKD2 with WT Abl resulted in a potent phosphorylation of Tyr-717 in the absence of H_2_O_2_ (Fig. 2b). We could confirm the involvement of Abl in PKD2 Tyr-717 phosphorylation using siRNA targeted against Abl, which also resulted in a strong decrease in Tyr-717 phosphorylation following oxidative stress (Fig. 2c). Direct phosphorylation at Tyr-717 by Abl was confirmed in an *in vitro* kinase assay (Fig. 2d). Collectively these results show that Abl acts as an upstream kinase for Tyr-717 under oxidative stress conditions.

### Phosphorylation of PKD2 on tyrosine residues is dependent on phosphorylation of activation loop serine residues

**Figure 3:**
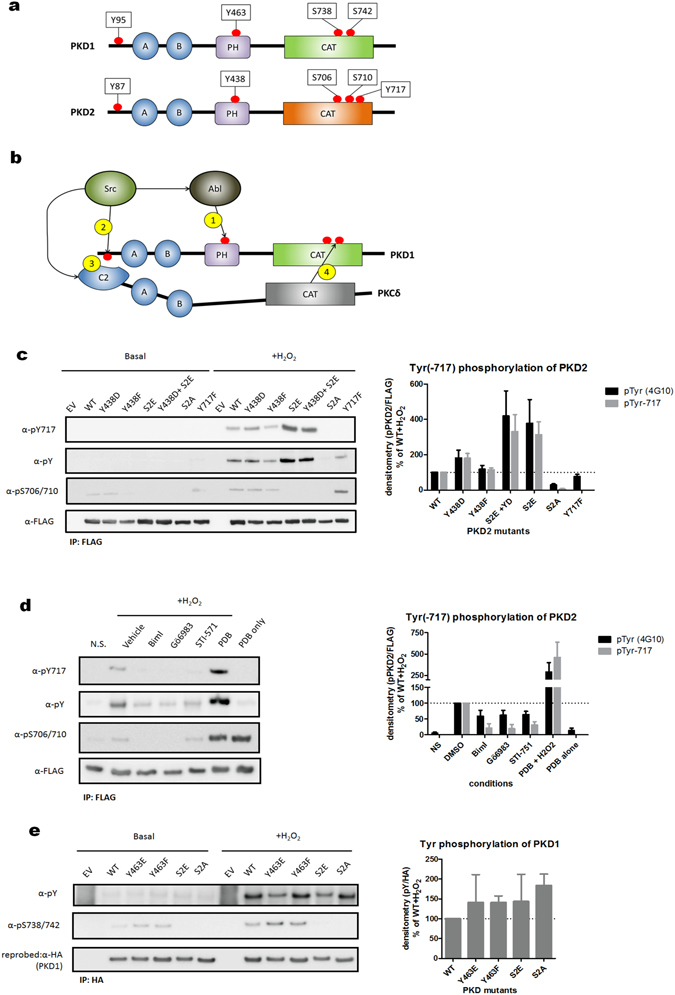
The mechanism underlying tyrosine phosphorylation of PKD2 in oxidative stress is different from that of PKD1. (**a**) Graphical representation of the different phosphorylation sites covered in this study in PKD1/2 (**b**) Activation model of PKD1 in oxidative stress (for details see text). (**c**) Tyrosine phosphorylation of PKD2 is dependent on activation loop Ser-706/710 phosphorylation. HEK293 cells were transfected with wild type (WT) PKD2 or the indicated mutants (S2E = S706/710E, S2A = S706/710 A). 48 h after transfection, cells were stimulated with H_2_O_2_ (10 mM, 10 min) and PKD2 was precipitated from the cells using FLAG antibody. Immunoprecipitates were subjected to western blot and probed with the indicated antibodies. Quantification of three independent experiments is shown. Western blots were cropped for clarity; uncropped images can be found in Supplementary Fig. S12. (**d**) Effect of PKC inhibitors and PDB on tyrosine phosphorylation of PKD2. HEK293 cells were transfected with FLAG-PKD2. 48 h after transfection, cells were treated with inhibitors (3 µM of BimI, 10 µM of Gö6983 or 5 µM STI571) or activator (500 nM PDB) and subsequently stimulated with H_2_O_2_ (10 mM, 10 min). Immunoprecipitates were subjected to western blot and probed with the indicated antibodies. Quantification of three independent experiments is shown. Western blots were cropped for clarity; uncropped images can be found in Supplementary Fig. S13. (**e**) The activation mechanism of PKD1 in oxidative stress differs from that of PKD2. HEK293 cells were transfected with wild type (WT) PKD1 or the indicated mutants (S2E = S738/742E, S2A = S738/742A). 48 h after transfection, cells were stimulated with H_2_O_2_ (10 mM, 10 min) and PKD1 was precipitated from the cells using HA antibody. Immunoprecipitates were subjected to western blot and probed with the indicated antibodies. Quantification of three independent experiments is shown. Western blots were cropped for clarity; uncropped images can be found in Supplementary Fig. S14.

PKDs are regulated by multi-site phosphorylation (Fig. 3a). Therefore, and in order to further elucidate the role of Tyr-717 phosphorylation in the regulation of PKD2 in oxidative stress conditions, we examined the interdependency of Tyr-717 phosphorylation on other known Ser/Tyr phosphorylation sites in PKD2.

For PKD1, activation during oxidative stress requires a hierarchical multistep phosphorylation sequence. First, Tyr-463 in the PH domain is phosphorylated, which promotes N-terminal Tyr-95 phosphorylation, allowing PKCδ to dock to the N-terminal phospho-tyrosine motif to subsequently phosphorylate the activation loop at Ser-738/742 (schematically represented in Fig. 3b).

We assessed the dependency of Tyr-717 phosphorylation in PKD2 on Tyr-438 phosphorylation (homologous to Tyr-463 in PKD1, the first step in the PKD1 activation model) and Ser-706/710 phosphorylation (the last step in the PKD1 activation model) using phosphomimetic and non-phosphorylatable mutants of these sites.

Interestingly, we observed that Tyr-717 phosphorylation was partly dependent on Tyr-438 phosphorylation, with some increase in the case of a phosphomimetic substitution, but no decrease in Tyr-717 phosphorylation in a Y438F mutant (Fig. 3c).

In contrast however, we observed a strong dependency of Tyr-717 phosphorylation on activation loop Ser-706/710 phosphorylation. Indeed, when a PKD2 mutant with Ser-706/710-Glu substitutions was retrieved from cells stressed with H_2_O_2_, we observed a potent (about 4-fold) increase in Tyr-717 phosphorylation compared to WT. Conversely, a Ser-706/710-Ala mutant showed complete loss of Tyr-717 phosphorylation (Fig. 3c). Furthermore, similar effects were observed for overall tyrosine phosphorylation of PKD2, as demonstrated with a general phospho-tyrosine antibody (Fig. 3c). It thus became clear that tyrosine phosphorylation of PKD2 is dependent on activation loop Ser-706/710 phosphorylation.

To further substantiate these findings, we modulated PKC activity in oxidative stress conditions and probed for the effect on Tyr-717 and overall tyrosine phosphorylation of PKD2. We used both PKC inhibitors (BimI and Gö6983) and the PKC activator PDB. Treatment with BimI and Gö6983 resulted in an expected decrease of Ser-706/710 phosphorylation (Fig. 3d), but also in a decrease in Tyr-717 and overall tyrosine phosphorylation (Fig. 3d). Conversely, when cells were pretreated with PDB and subsequently exposed to oxidative stress, we noticed a marked increase in Tyr-717 and overall tyrosine phosphorylation. Treatment with PDB alone did not result in any tyrosine phosphorylation of PKD2 while potently stimulating Ser-706/710 phosphorylation (Fig. 3d). These results further substantiate the conclusion that activation loop Ser-706/710 phosphorylation potentiates tyrosine phosphorylation of PKD2 in oxidative stress conditions.

In agreement with previously published data^46, 47^, we did not observe a dependency of Tyr phosphorylation on the activation loop Ser phosphorylation state in PKD1, since both Ser-738/742-Glu and Ala mutants had similar levels of overall Tyr phosphorylation (Fig. 3e). This indicates that these enzymes, though closely related, display divergent activation mechanisms in oxidative stress conditions.

### The interaction between PKD2 and PKCδ is independent of N-terminal Tyr-87 phosphorylation

In the activation model for PKD1 in oxidative stress, Tyr-95 phosphorylation by Src creates a docking site for the C2 domain of PKCδ which in turn phosphorylates Ser-738/742. In contrast, in PKD2 Ser-706/710 phosphorylation primes for all PKD2 tyrosine phosphorylation during oxidative stress, since the PKD2 S706/S710A mutation completely abolishes subsequent tyrosine phosphorylation of PKD2. Hence, this would imply that PKCδ can efficiently bind and phosphorylate non-tyrosine phosphorylated PKD2 in oxidative stress.

**Figure 4:**
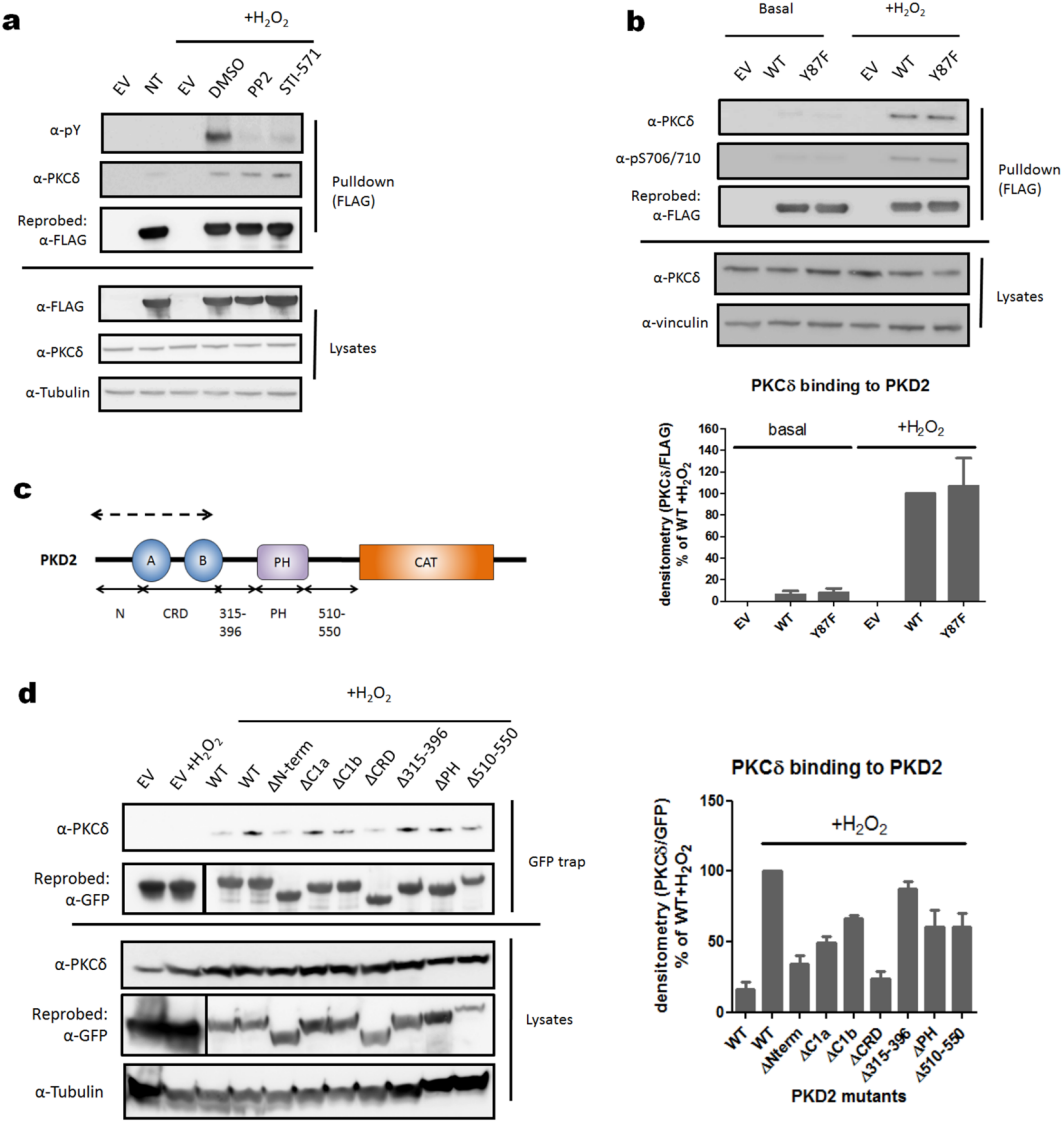
Different binding mode of PKCδ to PKD1 versus PKD2 in oxidative stress conditions. (**a**) Tyrosine kinase inhibition does not have an effect on the association between PKCδ and PKD2 during oxidative stress. HEK293 cells were transfected with wild type (WT) PKD2. 48 h after transfection, cells were treated with PP2 (10 µM) or STI-571 (5 µM) and subsequently stimulated with H_2_O_2_ (10 mM, 10 min) and PKD2 was precipitated from the cells using FLAG antibody. Immunoprecipitates were subjected to western blot and probed with the indicated antibodies. Western blots were cropped for clarity; uncropped images can be found in Supplementary Fig. S15. (**b**) Tyr-87 phosphorylation is not required for the association between PKD2 and PKCδ. HEK293 cells were transfected with wild type (WT) PKD2 or the indicated mutants. 48 h after transfection cells were stimulated with H_2_O_2_ (10 mM, 10 min) and PKD2 was precipitated from the cells using FLAG antibody. Immunoprecipitates were subjected to western blot and probed with the indicated antibodies. Quantification of three independent experiments is shown. Western blots were cropped for clarity; uncropped images can be found in Supplementary Fig. S16. (**c**) Schematic representation of PKD2 deletion mutants used for mapping the PKCδ binding domain. Deleted regions are denoted with full arrows. The proposed PKCδ binding region is indicated by a dotted arrow. (**d**) The N-terminal AP region and CRD of PKD2 are necessary for PKCδ interaction. HEK293 cells were transfected with wild type (WT) PKD2 or the indicated mutants. 48 h after transfection, cells were stimulated with H_2_O_2_ (10 mM, 10 min) and PKD2 was precipitated from the cells via GFP trap. Immunoprecipitates were subjected to western blot and probed with the indicated antibodies. Quantification of three independent experiments is shown. Western blots were cropped for clarity; uncropped images can be found in Supplementary Fig. S17.

Therefore, we probed for the association between PKCδ and PKD2 in oxidative stress in presence or absence of tyrosine kinase inhibitors PP2 (Src) or STI-751 (Abl). We could indeed show that this interaction is not affected by tyrosine kinase inhibition (Fig. 4a). To further verify that N-terminal Tyr-87 phosphorylation in PKD2 is not required for PKCδ binding and Ser-706/710 phosphorylation, we probed for PKCδ interaction with a non-phosphorylatable PKD2 Y87F mutant. When this mutant was precipitated from cells stressed with H_2_O_2_, we observed similar PKCδ binding to WT and Y87F kinase and Ser-706/710 phosphorylation was not affected (Fig. 4b). This indicates that, for PKD2 at least, ROS-stimulated interaction with PKCδ does not require Tyr-87 phosphorylation.

We next wondered which (additional) motifs could favor interaction of PKD2 with PKCδ by deleting different regions in the regulatory domain of PKD2 (Fig. 4c). We observed decreased PKCδ interaction when we deleted the N-terminal alanine/proline rich region of PKD2, but also when we deleted the cysteine rich domain (CRD) containing the two C1 domains (Fig. 4d). Deletion of one C1 domain had only partial effects on PKCδ binding (Fig. 4d).

### The three PKD isoforms are differentially phosphorylated on Tyr in the activation segment

**Figure 5:**
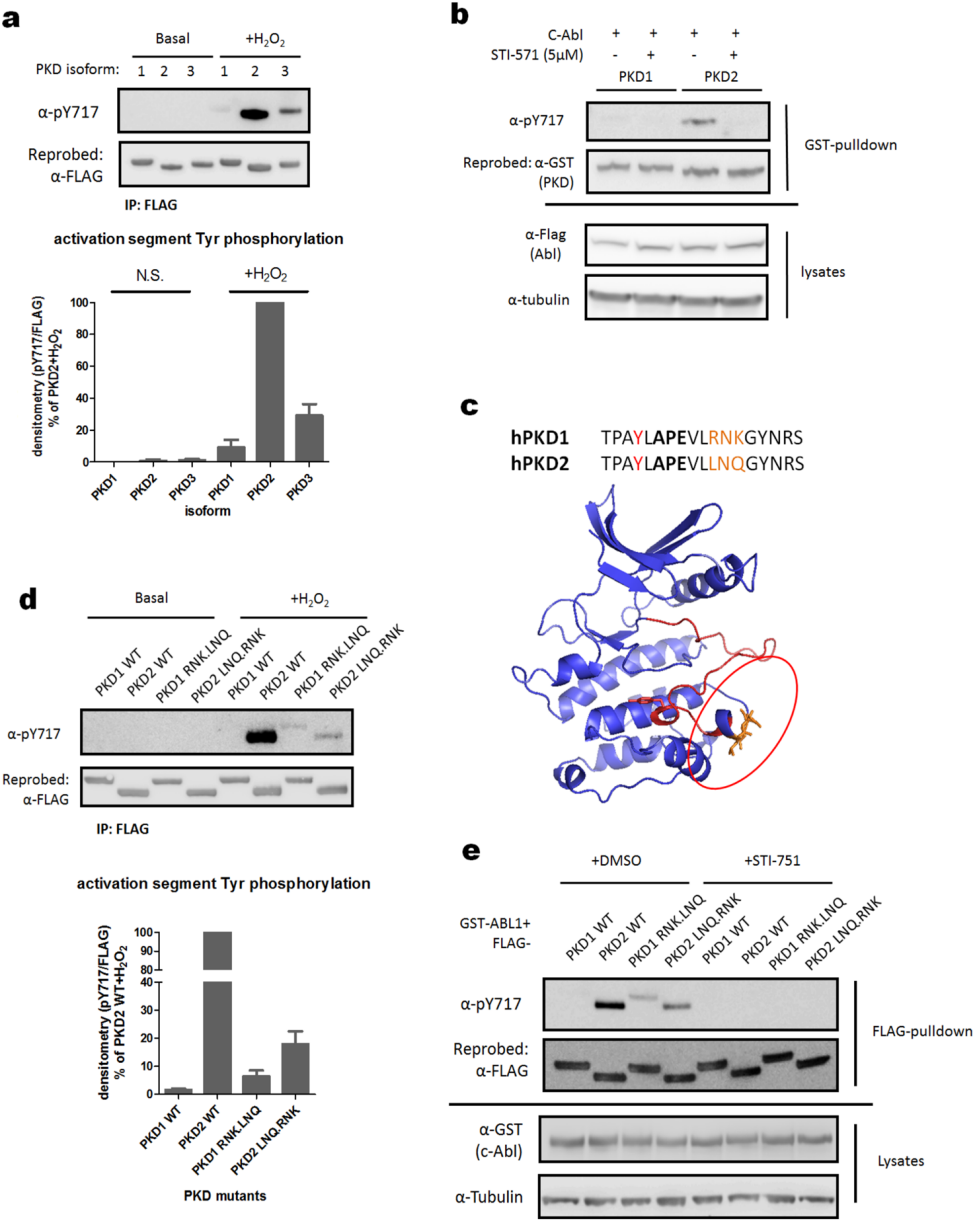
The PKD2-specific LNQ motif in the activation segment underlies the differential phosphorylation of PKD isoforms at Tyr in the P + 1 loop during oxidative stress. (**a**) The three hPKD isoforms are not equally phosphorylated at Tyr in the activation segment upon oxidative stress. HEK293 cells were transfected with FLAG-tagged wild type (WT) PKD1/2/3. 48 h after transfection cells were stimulated with H_2_O_2_ (10 mM, 10 min). FLAG immunoprecipitates were subjected to western blot and probed with the indicated antibodies. Quantification of three independent experiments is shown. Western blots were cropped for clarity; uncropped images can be found in Supplementary Fig. S18. (**b**) Abl does not phosphorylate PKD1 at the activation segment Tyr residue. HEK293 cells were transfected with wild type (WT) GST-PKD1/2. 48 h after transfection, cells were pretreated with STI-571 (5 µM) or vehicle and PKD1/2 were precipitated from the cells using Glutathione-sepharose beads. Samples were subjected to western blot and probed with the indicated antibodies. Western blots were cropped for clarity; uncropped images can be found in Supplementary Fig. S19. (**c**) Location of the divergent PKD1/2 sequence in the primary and tertiary structure (PKD2 homology model generated by the Phyre2 server). (**d**) Swapping the RNK motif of PKD1 with the LNQ motif in PKD2 results in altered activation segment Tyr phosphorylation. HEK293 cells were transfected with wild type (WT) PKD1/2 or the exchange mutants. 48 h after transfection, cells were stimulated with H_2_O_2_ (10 mM, 10 min) or left unstimulated. FLAG immunoprecipitates were subjected to western blot and probed with the indicated antibodies. Quantification of three independent experiments is shown. Western blots were cropped for clarity; uncropped images can be found in Supplementary Fig. S20. (**e**) Swapping the RNK motif of PKD1 with the LNQ motif in PKD2 results in altered activation segment Tyr phosphorylation by Abl. HEK293 cells were co-transfected with FLAG-tagged wild type (WT) PKD1/2 or the exchange mutants and GST-tagged Abl. Prior to lysis, cells were treated with STI-571 (5 µM) or DMSO. FLAG immunoprecipitates were subjected to western blot and probed with the indicated antibodies. Western blots were cropped for clarity; uncropped images can be found in Supplementary Fig. S21.

Since the activation segment (containing Tyr-717) is entirely conserved in all three PKD isoforms, the antibody we raised against the phospho-Tyr-717 site of PKD2 is fully cross-reactive to the other isoforms. Strikingly, we observed that the degree of Tyr phosphorylation in the YxAPE motif in response to oxidative stress is significantly different between the isoforms. Indeed, PKD2 is most abundantly phosphorylated, with 70% less phosphorylation in PKD3 and 90% less in PKD1 when compared to PKD2 (Fig. 5a). To elucidate whether this difference is due to a signaling pathway specifically targeting PKD2 during oxidative stress, or to the fact that Abl is incapable to phosphorylate PKD1, we co-expressed GST-tagged PKD1 and PKD2 with FLAG-tagged Abl. As expected, we could detect phosphorylation of PKD2 at Tyr-717, which was completely reversed by addition of the Abl inhibitor STI-571. However, Abl was unable to phosphorylate PKD1 at Tyr-749, indicating that Abl cannot efficiently phosphorylate PKD1 *in cellulo* (Fig. 5b). It is known that PKD1/2 and Abl do not form a stable complex during immunoprecipitation^46^, and we were also not able to show such an interaction (data not shown). Since PKD1 is known to be phosphorylated by Abl in the PH domain, but is not phosphorylated in the P + 1 loop, we reasoned that a local molecular difference between PKD1 and PKD2 might contribute to their differential phosphorylation in the activation segment. Interestingly, while the kinase domains of hPKD1 and hPKD2 are 95% identical, the transition region between subdomain VIII (which ends with the APE motif) and subdomain IX is different between the two isoforms. Indeed, PKD1 contains a positively charged ^764^RNK^766^ motif, while PKD2 contains a non-charged, partially hydrophobic ^724^LNQ^726^ motif in this region (Fig. 5c). The region C-terminal of the APE motif together with the αG helix is important for interactions of many protein kinases with upstream kinases, substrates, regulatory proteins, and phosphatases^56–59^. We therefore swapped the three differential amino-acids between PKD1 and PKD2 and probed these exchange mutants for Tyr phosphorylation in the activation segment upon oxidative stress. Interestingly, we observed a strong decrease of Tyr-717 phosphorylation in PKD2 when its ^724^LNQ^726^ motif was swapped with ^764^RNK^766^ (Fig. 5d). Conversely, we also observed increased Tyr-749 phosphorylation of PKD1 when its ^764^RNK^766^ motif was swapped with ^724^LNQ^726^, albeit rather moderately (Fig. 5d). Similar effects were also observed upon co-expression of Abl with PKD2 and the exchange mutants (Fig. 5e). These data indicate that the LNQ motif is essential, but not sufficient for activation segment Tyr phosphorylation of PKD1/2 by Abl.

### Activation segment Tyr phosphorylation increases PKD activity towards peptide substrate

The tyrosine residue identified as a phospho-acceptor site in this study resides in the P + 1 loop of the activation segment of the kinase (Fig. 1a). Phosphorylation of this residue thus may alter the interaction with and the activity towards substrates.

To test for possible differences in substrate specificity we performed a peptide array analysis, in which we compared PDB-stimulated (containing no pTyr) and H_2_O_2_-stimulated (Tyr-phosphorylated) PKD2. However, we observed no obvious consistent differences in amino-acid preference between the two conditions (see Supplementary Fig. S2), indicating that Tyr-717 phosphorylation does not strongly affect substrate specificity.

To probe for potential effects of Tyr-717 phosphorylation on activity, we investigated the kinetics of the different PKD isoforms and mutants towards the model substrate Syntide-2 (Syn-2).

**Figure 6:**
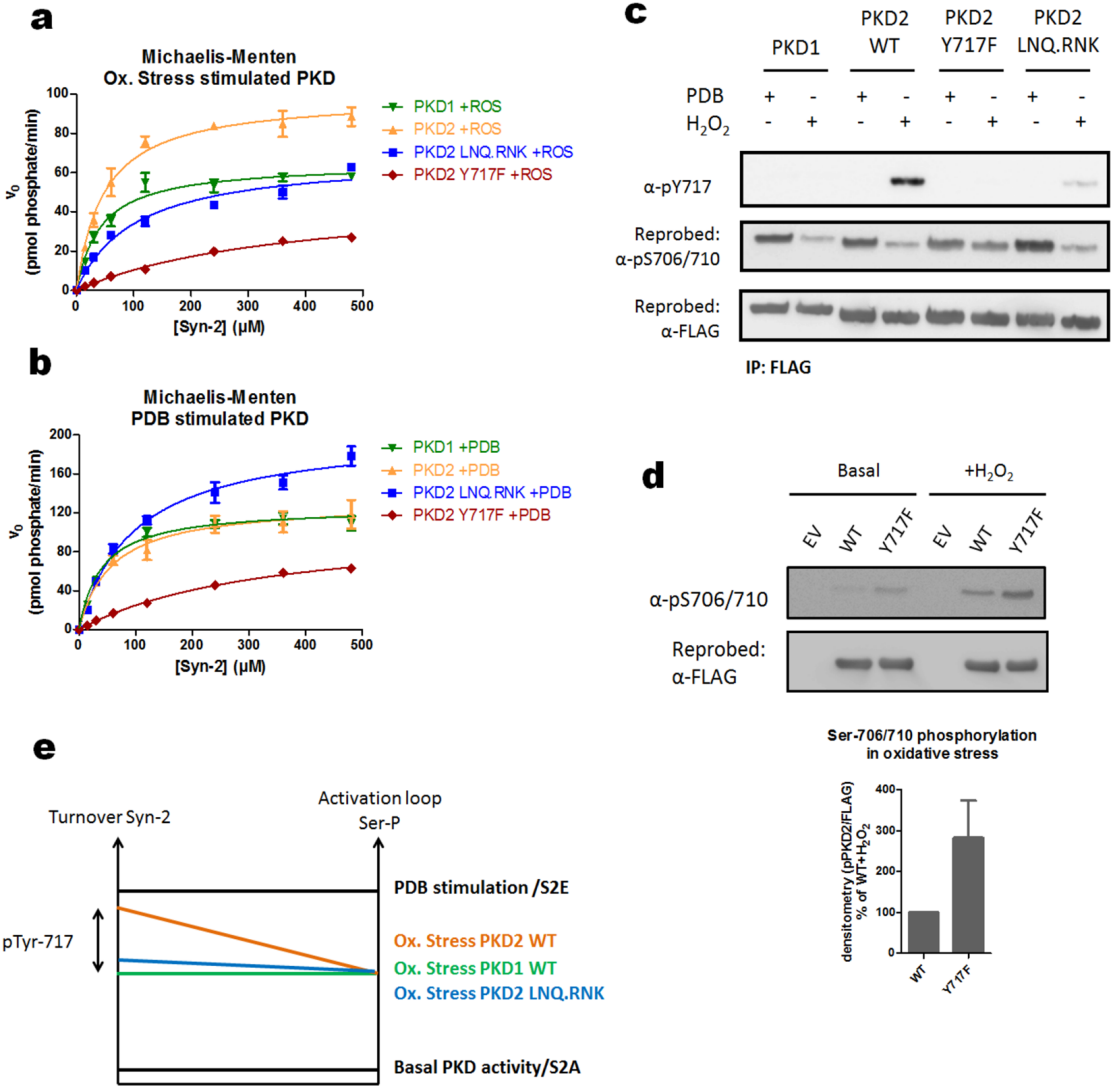
Oxidative stress-induced Tyr717 phosphorylation of PKD2 increases kinase activity towards syntide-2. (**a**) Kinetics of WT PKDs and the indicated mutants after activation by oxidative stress. FLAG-PKDs were purified from HEK293 cells after stimulation with H_2_O_2_ (10 mM, 10 min). Protein concentration and purity (100%) was analyzed side by side by densitometry of a coomassie stained SDS-polyacrylamide gel using a BSA standard. Michaëlis-Menten kinetics for Syn-2 phosphorylation by each protein was followed in a radiometric kinase assay (see Materials and methods). (**b**) FLAG-PKDs were purified from HEK293 cells after stimulation with PDB (500 nM, 15 min). The purified proteins were subjected to the same analysis as in 6a. (**c**) Activation segment Ser and Tyr phosphorylation of stimulated enzymes. The purified enzymes described in 6a and 6b were analyzed for Ser and Tyr phosphorylation in the activation segment via Western Blotting with the indicated antibodies. Western blots were cropped for clarity; uncropped images can be found in Supplementary Fig. S22. (**d**) PKD2 Y717F displays higher activation loop Ser phosphorylation compared to PKD2 WT. HEK293 cells were transfected with wild type (WT) PKD2 or an Y717F mutant. 48 h after transfection, cells were stimulated with H_2_O_2_ (10 mM, 10 min) and PKD2 was precipitated from the cells using FLAG antibody and subsequently probed for Ser-706/710 phosphorylation. Quantification of three independent experiments is shown. Western blots were cropped for clarity; uncropped images can be found in Supplementary Fig. S23. (**e**) Correlations between activation loop Ser phosphorylation and kinase activity towards peptide substrate are indicated by full lines. Pools of PKD1 and PKD2 WT enzymes harbor similar levels of activation loop Ser phosphorylation in oxidative stress, but PKD2 has higher activity towards Syn-2, which is due to Tyr-717 phosphorylation.

Comparing PKD activities in oxidative stress conditions, we observed similar K_m_ values for PKD1 and PKD2, but interestingly, k_cat_ values for PKD2 were about 1.5 fold higher than for PKD1 (Fig. 6a and Table 1). This discrepancy was only observed in oxidative stress stimulated enzymes, since in PDB stimulated conditions both isoforms share similar kinetics (Fig. 6b and Table 1).

**Table 1:** Kinetic parameters for the different PKD preparations, values shown are mean ± SD.

parameter	K_m_ (µM)		V_max_ (pmol phosphate/min)		k_cat_ (pmol phosphate/min/pmol PKD)	
Conditions:	PDB	Ox. stress	PDB	Ox. stress	PDB	Ox. stress
PKD1 WT	39.8 ± 5.4	42.5 ± 4.2	125.8 ± 6.4	64.87 ± 2.5	128.4	66.2
PKD2 WT	57.5 ± 11.7	47.8 ± 7.4	130.8 ± 6.6	98.91 ± 3.55	126.9	96
PKD2 Y717F	337 ± 62.7	371.1 ± 10.6	109.2 ± 55.5	49.19 ± 3.98	106	47.8
PKD2 LNQ.RNK	97.7 ± 12.8	101.2 ± 8.8	203.4 ± 15.6	68.29 ± 3.52	197.5	66.3

To examine whether this difference in activity could be due to Tyr-717 phosphorylation in PKD2, we made use of the aforementioned PKD2 LNQ.RNK exchange mutant, which shows a strong reduction in Tyr-717 phosphorylation compared to WT PKD2, without aberrations in activation loop Ser-706/710 phosphorylation (Fig. 6c). Interestingly, this PKD2 mutant follows similar kinetics as wild-type (WT) PKD1 during oxidative stress (albeit with slightly higher K_m_ values) indicating that Tyr-717 phosphorylation is responsible for the increase in k_cat_ for PKD2 (Fig. 6a and Table 1). In PDB stimulated conditions, this mutant remarkably shows higher activity than WT PKD1/2, which can be correlated to increased PDB-induced activation loop Ser-706/710 phosphorylation, for hitherto unknown reasons (Fig. 6b,c and Table 1).

Our kinetic analysis also indicates that a Tyr to Phe substitution is not an adequate non-phosphorylatable mutant, despite its use in several other studies (see discussion). Indeed, this substitution results in drastically increased K_m_ and lowered k_cat_ levels compared to WT PKD2, even in PDB stimulated conditions, where Tyr-717 is not phosphorylated (Fig. 6a,b and Table 1). Interestingly, while the Y717F mutant displays strongly impaired activity, activation loop Ser-706/710 phosphorylation is higher than in WT under oxidative stress (Fig. 6c and d). This indicates that this mutation possibly alters the activation segment conformation/flexibilty, increasing accessibility for upstream PKC, while it has an incorrect conformation for optimal catalysis.

**Figure 7:**
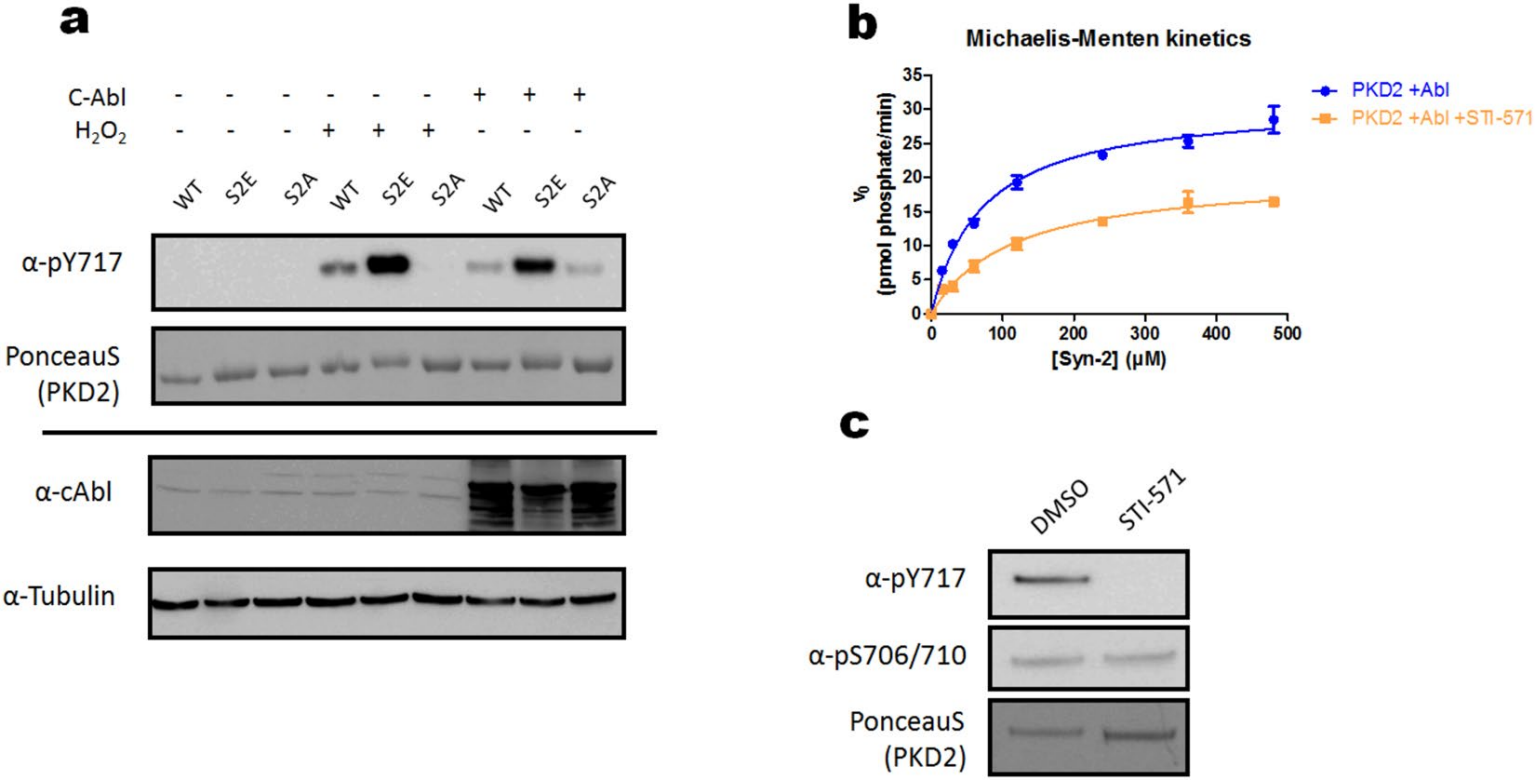
Tyr phosphorylation by Abl results in an increase in k_cat_ independent of the activation loop Ser phosphorylation state. (**a**) Tyr-717 phosphorylation of a PKD2 S706/710A mutant can occur upon co-expression with Abl. HEK293 cells were transfected with FLAG-tagged wild type (WT) PKD2 or the indicated mutants alone or in combination with GST-tagged Abl. 48 h after transfection, cells were stimulated with H_2_O_2_ (10 mM, 10 min) or left unstimulated and PKDs were precipitated from the cells using FLAG antibody. Immunoprecipitates were subjected to western blot and probed with the indicated antibodies. Western blots were cropped for clarity; uncropped images can be found in Supplementary Fig. S24. (**b**) Kinetics of PKD2 co-expressed with Abl with and without addition of STI-571 (5 µM) before cell lysis. Protein concentration and purity (100%) was analyzed side by side by densitometry of a coomassie stained SDS-polyacrylamide gel using a BSA standard. Michaëlis-Menten kinetics for Syn-2 phosphorylation by each protein was followed in a radiometric kinase assay (see Materials and methods). (**c**) Analysis of Ser-706/710 and Tyr-717 phosphorylation of PKD2 purified from cells co-expressing Abl, with and without addition of STI-571. The purified enzymes described in 7b were analyzed for Ser and Tyr phosphorylation in the activation segment via Western Blotting with the indicated antibodies. Western blots were cropped for clarity; uncropped images can be found in Supplementary Fig. S25.

To directly assess how Tyr-717 phosphorylation affects PKD2 activity, we purified PKD2 from unstimulated cells co-expressing Abl. It should be noted that in conditions of high-level expression, (unstimulated) Abl can phosphorylate PKD2 independent of activation loop Ser phosphorylation, although still preferring Ser-phosphorylated species as indicated by a PKD2 S706/710E mutant (Fig. 7a). This is in contrast to oxidative stress conditions, where endogenous levels of activated Abl are incapable of phosphorylating a PKD2 S706/S710A mutant (Fig. 7a, lane 6 and Fig. 3c). Kinetic analysis of purified PKD2 from Abl overexpressing cells treated with or without STI-571 revealed an increase in k_cat_ values for PKD2 phosphorylated at Tyr-717 by about 1.5 fold compared to a non-Tyr phosphorylated species (Fig. 7b and Table 2). This increase is independent of the activation loop Ser phosphorylation state, which is at similar (basal) levels in both conditions (Fig. 7c). In absolute terms, however this stimulatory effect is quite small compared to the stimulatory effect under oxidative stress when Ser-706/710 phosphorylation also increases (compare Abl-phosphorylated PKD2 k_cat_ = 32.6 to Ox. stress stimulated PKD2 k_cat_ = 96 pmol phosphate/min/pmol PKD).

**Table 2:** Kinetic parameters for the different PKD preparations, values shown are mean ± SD.

	K_m_ (µM)	V_max_ (pmol phosphate/min)	k_cat_ (pmol phosphate/min/pmol PKD)
PKD2 + Abl	71.69 ± 8.3	31.78 ± 1.063	32.6
PKD2 + Abl + STI-571	114.5 ± 18.45	20.59 ± 1.1531	21

### Tyr-717 phosphorylation does not affect NF-κB signaling by PKD2

**Figure 8:**
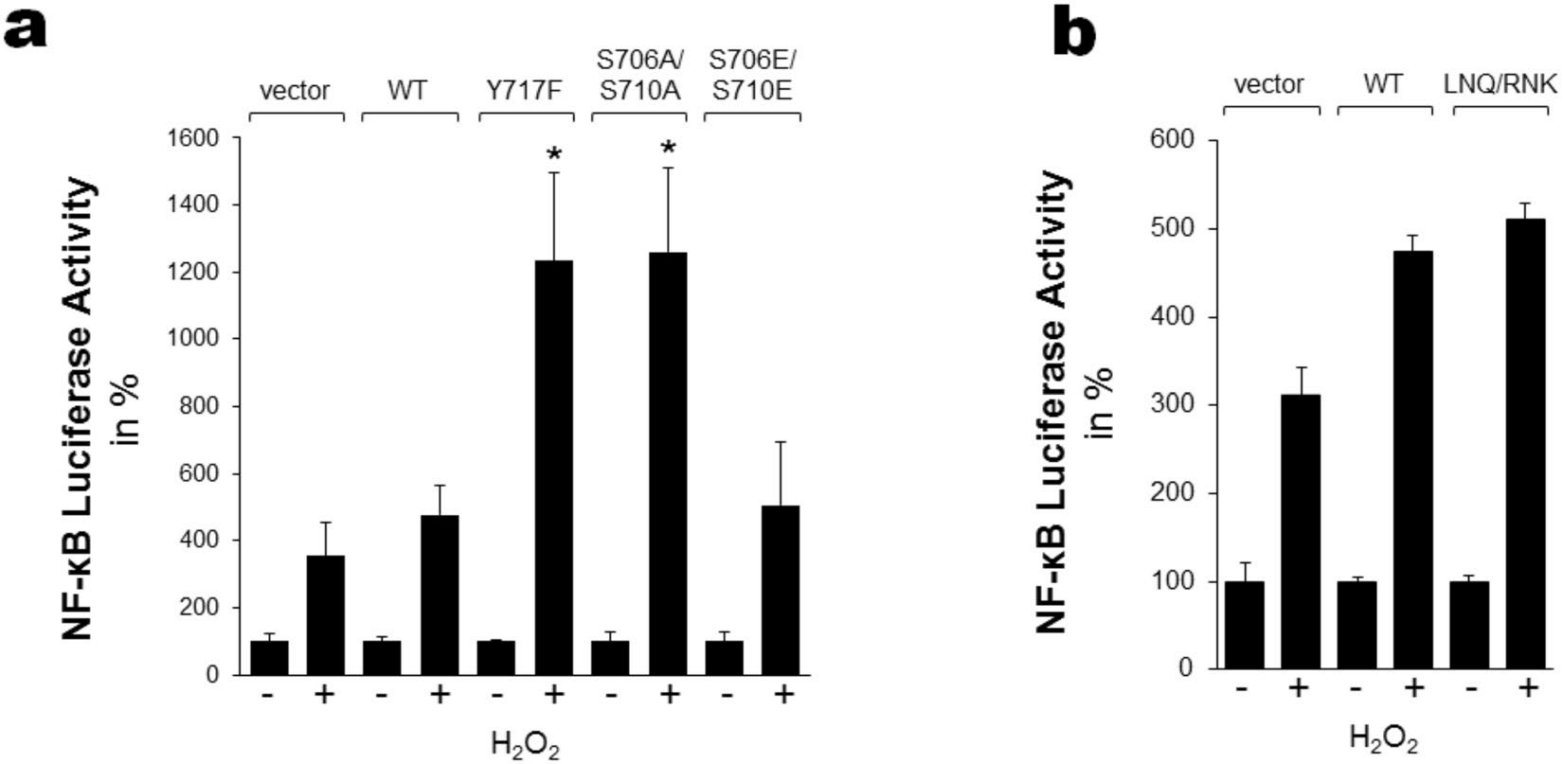
Signaling to NF-κB by different PKD2 mutants in oxidative stress conditions. (**a**) HeLa cells were co-transfected with control vector, indicated PKD2 expression constructs and NF-κB-luciferase and β-Galactosidase reporters. 4 hours after transfection, cells were stimulated with 500 µM H_2_O_2_. 18 hours after stimulation cells were lysed and reporter gene assays performed. Shown is the relative increase between untreated and treated sample. The asterisk indicates statistical significance (unpaired *t* test); Y717F: p = 0.0002; S706A/S710A: p = 0.0001. (**b**) HeLa cells were co-transfected with control vector, PKD2 wildtype or LNQ.RNK expression constructs and NF-κB-luciferase and β-Galactosidase reporters. 4 hours after transfection, cells were stimulated with 500 µM H_2_O_2_. 18 hours after stimulation, cells were lysed and reporter gene assays performed. Shown is the relative increase between untreated and treated sample.

Since PKDs are known to signal to NF-κB in oxidative stress, we wondered whether the phosphorylation of PKD2 on its regulatory sites, including Tyr-717, might influence the signaling output. Therefore, we tested WT PKD2 and several mutants for their ability to signal to NF-κB via a luciferase reporter assay (Fig. 8a). WT PKD2 displays an approximate 1.5 fold increase in NF-κB activity, but interestingly, strong potentiating effects are observed with S706/710 A and Y717F mutants. This could be due to loss of Tyr-717 phosphorylation, since both Y717F and S706/710A cannot be phosphorylated at Tyr-717 (Fig. 3c), but it could also be an effect of PKD inactivation, since a S706/710 A mutant is inactive, and we have shown here that a Y717F mutant is catalytically impaired (Fig. 6a,b). To discriminate these effects we used the PKD2 LNQ.RNK exchange mutant, which is still active, but shows strongly reduced Tyr-717 phosphorylation. This mutant supported levels of NF-κB reporter activation equivalent to WT PKD2 (Fig. 8b), indicating that effects observed with S706/710A and Y717F mutants are likely due to inactivation of PKD2 rather than blocking Tyr-717 phosphorylation. Consistent with this idea, increasing PKD2 activity in oxidative stress does not affect PKD2 signaling to NF-κB, as shown with a S706/710E mutant (Fig. 8a).

## Discussion

In this work, we show that PKD2 can be phosphorylated in oxidative stress conditions in the P + 1 loop of the kinase activation segment at a highly conserved Tyr residue just N-terminal of the APE motif.

This Tyr residue shows very high conservation among Ser/Thr kinases, with Tyr occurring in most (73%) cases, followed by Phe (15%) and Trp (11%). Despite the high conservation of the Tyr residue in the P + 1 loop, there are only a few reports of phosphorylation in the YxAPE motif in other kinases. For example, phosphorylation is also reported for PKCδ in oxidative stress conditions^58, 60^ and in EGF signaling for PKCε, PKCζ and PKB^8, 61, 62^. Also, the residue was implicated in activation of PKB downstream of RET/PTC^22^. IKKβ has also been reported as a kinase regulated by this site in TNF signaling to NF-κB^6, 63, 64^. Recently, ERK has been shown to be phosphorylated *in vitro* at this residue by MEK1^65^. Chk2 is also phosphorylated at this residue after irradiation^12^. All these studies make use of Tyr to Phe substitutions as non-phosphorylatable mutants, and one must be cautious to not over-interpret functional results with these mutants. This is especially evident in our NF-κB assays, where we observed a strong increase in signaling with the Y717F mutant, but not with the LNQ.RNK exchange mutant, which causes a disruption of Tyr-717 phosphorylation without directly affecting the phosphoacceptor. These misrepresentative results stem from the fact that a Tyr to Phe substitution results in drastic effects on kinase activity, with about a 7-fold increase in K_m_ values for Syn-2 phosphorylation, occurring even in PDB stimulated conditions where WT PKD2 is not phosphorylated on Tyr-717. A similar increase in K_m_ has been shown for an analogous Y204F substitution in PKA, which has not yet been identified to be phosphorylated at this residue^66^. This indicates that for Tyr containing kinases the hydroxyl group is of critical importance for their activity. It would be interesting to determine why Phe containing enzymes do not have this requirement.

Interestingly, while a Y717F substitution in PKD2 resulted in impaired kinetics, activation loop Ser-706/710 phosphorylation is even higher than in WT PKD2. This is possibly due to alterations in the conformation of the activation segment, facilitating phosphorylation of the Ser residues by PKCδ. These observations emphasize the fact that activation loop phosphorylation cannot always be used as a readout for kinase activity. This was also shown in a previous study using the allosteric PKD inhibitor CID-755673, where PKD was shown to display elevated activation loop Ser phosphorylation upon inhibition^67^.

Strikingly, the phosphosite database reports phosphorylation at the YxAPE motif in more than 70 kinases (combining human, rat and mouse proteomes), suggesting that this phenomenon might be more widespread than was previously appreciated (www.phosphosite.org and Supplementary Table S3).

We observed differential phosphorylation levels of the YxAPE Tyr after oxidative stress within the PKD family, despite 100% conservation of this motif in all three PKD isoforms. One of the molecular determinants that we identified for the discrepant phosphorylation at this site is the loop between subdomain VIII and IX, just C-terminal of the APE motif. While the kinase domains of the three PKD isoforms share 90% sequence identity, there is remarkable divergence in this region, with a RNK motif in PKD1, a LNQ motif in PKD2 and a RSK motif in PKD3. Together with the αG helix this region is important for interactions with upstream kinases, substrates, regulatory proteins, and phosphatases^56–59^. When we swapped the LNQ motif of PKD2 with the RNK motif of PKD1, we observed a drastic decrease in Tyr-717 phosphorylation. This decrease is possibly due to the charge content in this loop in PKD1/3 that might be electrostatically unfavorable for interaction with Abl. Having established that the Y717F substitution is disruptive for PKD2 function, the mutant where this region was swapped between PKD1 and PKD2 was an ideal tool to study the effects of Tyr phosphorylation in the P + 1 loop on kinase activity. By following the kinetics of WT PKD1/2 and the PKD2 LNQ.RNK mutant, we were able to show that Tyr-717 phosphorylation can modulate the activity of PKD2 towards the model peptide substrate Syn-2. In particular, Tyr-717 phosphorylated PKD2 displays higher turnover compared to a non-phosphorylated species. Tyr phosphorylation in the P + 1 loop alters the charge, and possibly the conformation of the activation segment, which may enhance the release of phosphorylated substrate.

The differential regulation of PKD isoforms in oxidative stress extends beyond Tyr phosphorylation in the P + 1 loop. Our experiments also revealed that the activation mechanism of PKD isoforms is entirely different. While PKD1 has been shown to be dependent on Tyr-463 and Tyr-95 phosphorylation for activation loop Ser-738/742 phosphorylation, we do not see this dependency in PKD2. In fact, we see the opposite: activation loop Ser-706/710 phosphorylation primes for subsequent Tyr phosphorylation. PKD2 can also be phosphorylated at Tyr-438 in the PH domain, (corresponding to Tyr-463 in PKD1), for example in BCR-ABL^+^ cell lines and upon interferon stimulation of HeLa cells^54, 68^. While this residue is not implicated in the PKD2 activation mechanism, it may play a role in stabilization of the active conformation. The PKCδ–PKD2 interaction is not dependent on N-terminal Tyr-87 phosphorylation, whereas it has been shown to be highly dependent on Tyr-95 phosphorylation in PKD1^47^. PKD3 contains a Phe residue at this position, but nonetheless has recently been shown to be activated via oxidative stress in fibroblasts, which is reversible by treatment with the PKC inhibitor GF109203X^69^. This suggests that PKD3 can also be activated by PKCs in oxidative stress despite the absence of a pTyr in the N-terminus. It is likely that additional motifs are present in PKD2/3 that facilitate the interaction with upstream PKCs. For PKD2 we could show that the extreme N-terminal AP-rich region, as well as the CRD is important for a robust interaction. The different determinants for PKCδ binding could be explained by structural diversity, since this region (AP-rich region + CRD) is only 48% identical between the 3 isoforms.

The differential biochemical regulation of PKD isoforms in oxidative stress exposes a potential mechanism for functional divergence between the isoforms. Such divergence has been reported in restricted cases. For example, PKD1 and PKD2, but not PKD3 phosphorylate PI4KIIIβ^70^. Furthermore, PKD2, and not PKD1 specifically regulates MMP9 secretion^71^. It was also shown that in cardiomyocytes α1-ARs selectively activate PKD1 while PAR-1 and PDGFRs selectively activate PKD2^72^. PKD2 was specifically activated during murine myoblast differentiation^73^. While differential activation is well documented, it is unclear what molecular mechanisms underlie this isoform-specific regulation. Our current study provides biochemical evidence for isoform-specific regulation in oxidative stress conditions through differential phosphorylation of Tyr in the P + 1 loop.

Since oxidative stress induces signaling from PKDs to NF-κB, we wondered if the ROS-induced Tyr-717 phosphorylation in PKD2 might play a role in this pathway. Intriguingly, we found that, while WT PKD2 signals to NF-κB, signaling is highly potentiated with Y717F and S706/710A mutants (both of which cannot be phosphorylated at Tyr-717 but are also inactive). By using the PKD2 LNQ.RNK exchange mutant we found that the observed effects are not due to loss of Tyr-717 phosphorylation, but rather to an abolishment of PKD2 activity. We therefore conclude that PKD2 activity is counterproductive for its signaling output to NF-κB signaling, which is consistent with results obtained by the Seufferlein lab, where it was also shown that NF-κB signaling by PKD2 is potentiated by a kinase dead mutant^54^. Since Tyr-717 phosphorylation results in increased PKD2 activity, it consequently does not potentiate signaling to NF-κB. The effects we observed are in marked contrast with PKD1 signaling to NF-κB, where PKD activity is crucial for signaling output^48^. The final signaling output is likely determined by the differential regulation and different protein levels of PKD1 and PKD2, which is an intriguing question for further investigation. However, while Tyr-717 phosphorylation does not affect NF-κB signaling, it might differentially affect other pathways in oxidative stress.

In conclusion, in this paper we show differential phosphorylation of PKD isoforms at a highly conserved Tyr residue in the activation segment P + 1 loop, which modulates kinase activity towards peptide substrate. These results might have broader implications for our understanding of kinase regulation in general since the residue is highly conserved among Ser/Thr kinases and is found to be phosphorylated in more than 70 cases in proteomic studies.

## Methods

### cell culture, antibodies and chemicals

HEK293 and HeLa cell lines were grown in Dulbecco's modified eagle medium (DMEM) supplemented with 10% (v/v) Fetal Bovine serum (GE Healthcare, Little Chalfont, UK), 2 mM glutaMAX (ThermoFisher Scientific, Waltham, MA, USA), 100 U/ml Penicillin and 100 µg/ml Streptomycin (ThermoFisher scientific). Anti-GST, Anti-FLAG M2 antibody, HA antibody and agarose resins were purchased from Sigma (St. Louis, MO, USA). Glutathione sepharose 4B beads were from GE healthcare (Little Chalfont, UK). GFP trap beads were from Chromotek (Planegg, Germany). Anti-phosphotyrosine antibody (4G10) was from Millipore (Billerica, MA, USA), PKD anti-pSer-744/748 antibody, anti-PKCδ antibody, secondary HRP-linked goat anti-Rabbit and Horse anti-Mouse antibodies were from Cell Signaling Technologies (Beverly, MA, USA). Phorbol 12,13-dibutyrate (PDB), ATP, Neurotensin, Bradykinin, Lysophosphatidic acid, STI-751, PP2, DPH and Hydrogen peroxide 30% (v/v) were from Sigma (St. Louis, MO, USA). Gö 6983 was from Selleckchem (Munich, GE). Polyethyleneimine (PEI) was from Polysciences Inc. (Warrington, PA, USA).

### Oligonucleotides, plasmids and cloning

Plasmids encoding for FLAG-PKD2 WT (pdcDNA-FLAG-PRKD2) and GST-PKD2 (pDEST27-PRKD2) were generated via an LR recombination reaction (Invitrogen, Carlsbad, CA, USA) of pDONR223-PRKD2, genetically modified to contain a stopcodon (primers: FW: CAAGAAAGTTGGCTAGAGAACACTGATGCGCTCCG, RV: CGGAGCGCATCAGTGTTCTCTAGCCAACTTTCTTG). Mutagenesis of pDONR223-PRKD1/2 was carried out by site directed mutagenesis via the Quikchange kit (Agilent, Santa Clara, CA, USA) for the following mutants: Tyr717 to Phe (FW: GCACGCCGGCCTTCCTGGCACCC, RV: GGGTGCCAGGAAGGCCGGCGTGC), Tyr87 to Phe (FW: TTCCCTGAGTGTGGCTTCTTCGGCCTTTACG, RV: CGTAAAGGCCGAAGAAGCCACACTCAGGGAA), PKD1 RNK-LNQ substitution (FW: GAGCGATTGTAGCCCTGGTTTAGTAGGACCTCAGGAGCCAGGTAAG, RV: CTTACCTGGCTCCTGAGGTCCTACTAAACCAGGGCTACAATCGCTC), PKD2 LNQ-RNK substitution (FW: CGGTTGTAGCCCTTGTTGCGCAGCACCTCGGG, RV: CCCGAGGTGCTGCGCAACAAGGGCTACAACCG). Expression clones were generated via the LR reaction as for PKD2-WT. PdcDNA-FLAG-ABL1 and pDEST27-ABL1 were generated using the LR reaction using pDONR233-ABL1 as a template. Plasmids encoding GFP-PKD2 WT, ΔCRD, ΔC1a, ΔC1b, ΔPH, Δ315–396 and Δ510–550 were a gift from Thomas Seufferlein and have been described elsewhere^74, 75^. Plasmids encoding FLAG-PKD2.Y438F, Y438D, S706/710E and S706/710 A were also provided by the Seufferlein lab and have been described elsewhere^54^. Plasmids encoding HA-PKD1 WT, Y463D, Y463F, S738/742E and S738/742A were gifts from the Toker lab, and obtained via Addgene^50^. NF-κB-luciferase (NF-κB-LUC) and pCS2-(n) β-Galactosidase reporters have been described elsewhere^50^. For siRNA experiments we used the TriFECTa DsiRNA kit (IDT DNA, Leuven, BE).

### Transfections

Transfections of HEK293 cells were carried out with polyethyleneimine (PEI) at a 1:3 (m/m) plasmid/PEI ratio. For siRNA-mediated knockdown of Abl, cells were plated and transfected the next day with 10nM of control or antisense nucleotide using lipofectamine 2000 (Invitrogen, Carlsbad, CA, USA). Twenty-four hours later, a second transfection was carried out with pcDNA-FLAG-PRKD2 using PEI at a 1:3 (m/m) plasmid/PEI ratio. Cells were treated and lysed 48 h post-transfection.

### Antibody production

For the production of the site-specific phospho-antibody (PSSA) directed against phospho-Tyr-717, rabbits were immunized with KLH coupled peptide (CPApYLAPEV) using a standard immunization protocol. All animal procedures were done in accordance with KU Leuven - University of Leuven guidelines and regulations. Animal experiments were approved by the animal ethical committee (ECD) of the KU Leuven - University of Leuven (https://admin.kuleuven.be/raden/en/animal-ethics-committee) (project P146/2010). Antibodies were precipitated from the rabbit serum with 50% ammonium sulphate and dissolved in PBS (Harlow E. and Lane D., 1988, p. 298–299). Phospho-specific antibodies were purified sequentially on a non-phospho-peptide column and a phospho-peptide column. These were prepared by binding peptides to SulfoLink resin (Pierce) according to the protocol of the manufacturer (Thermo Fisher Scientific, Waltham, MA, USA). Antibodies were eluted using 100 mM Glycine pH 3.0 and immediately neutralized with 1 M Tris-HCl, pH 8. The specificity of the antibody was determined in an ELISA using BSA coupled phospho and non-phospho peptide and via immunoblotting.

### Mass-spectrometry analysis

To detect Tyr-717 phosphorylation after oxidative stress via mass-spectrometry, we performed a pull-down of GST-tagged PKD2 from HEK293 cells stimulated with H_2_O_2_ or left unstimulated. GST-PKD2 was eluted from glutathione-sepharose beads and subjected to TCA/aceton precipitation and trypsin digestion (10 µg modified trypsin (Promega) in 200 mM AmBic, 5% CH_3_CN, 0.1% RapiGest). Digested proteins were subjected to desalting on C18 Micro Spin Columns (Harvard Apparatus) and equal fractions were loaded on a hybrid quadrupole-orbitrap nano LC-MS system (QExactive, Thermo Fisher Scientific) using a data dependent analysis method. Number of ‘queries matched’ for the PKD2 protein was compared between conditions, and equal amounts of protein were subjected to phospho-enrichment on 50 µl IMAC bead suspension (Phos-Select, Sigma). The eluates were again desalted by C18 Micro Spin Columns and loaded on a QExactive LC-MS system using a targeted analysis method (tMS2). Data analysis was executed by using the MASCOT (Matrix Science) search engine together with the Proteome Discoverer 1.4 PhosphoRS 3.0 workflow. Data mining was done with XCalibur 3.0.63 Qual Browser and MZmine software. The phosphorylation site assignment was manually verified.

### Pulldowns and immunoprecipitations

Before lysis, cells were treated with the indicated compounds (see figures) for 20 min or left untreated. Lysis was done in 50 mM Tris, pH 7.4, 150 mM NaCl, 15 mM EDTA, 1% NP-40 supplemented with phosphatase inhibitors (Phosphostop, Roche, Germany), and protease inhibitors (cOmplete, Roche, Germany). Cell lysates were incubated with affinity beads for 2 hours at 4 °C while rotating (Glutathione Sepharose 4B (GE healthcare), GFP nanobody coated beads (Chromotek, Planegg, GE), anti-FLAG M2 beads (Sigma, St. Louis, MO, USA), anti-HA beads (Sigma, St. Louis, MO, USA)). Alternatively, for immunoprecipitation of endogenous PKD2, lysates were incubated with 2 µg anti-PKD2 antibody (Bethyl Laboratories, Montgomery, TX, USA) for 2 h, after which protein A beads were added for another hour. Next, the beads were washed twice with NENT500 (50 mM Tris, pH 7.4, 1 mM EDTA, 500 mM NaCl, 0.1% NP40, 25% glycerol) and once with NENT150 (cf. NENT500 but containing 150 mM NaCl). Alternatively, in co-IP experiments, three washes with NENT150 were performed. Elution was done in 50 mM Tris.HCl pH 7.4, 50 mM NaCl, 25% glycerol using competing peptide (FLAG/HA pulldowns), 20 mM glutathione (GST-pulldowns) or boiling in SDS sample buffer for 5 min at 95 °C.

### Activity assays and kinetics

For *In vitro* phosphorylation of PKD2 by Abl, purified FLAG-PKD2 (K580A mutant) was incubated with 50 ng of purified Abl (Carna Biosciences, Kobe, JP) and allowed to incubate for 30 min at 30 °C. Reactions were stopped by addition of SDS sample buffer. Subsequently, samples were boiled at 95 °C for 5 min prior to loading of an SDS-polyacrylamide gel.

For kinetic analysis of FLAG-PKD and its mutants, enzymes were purified from HEK293 cells as described above, but the NENT500 wash was substituted by a stringent NENT750 (50 mM Tris, pH 7.4, 1 mM EDTA, 750 mM NaCl, 0.1% NP40, 25% glycerol) wash. Protein purity (100%) and concentrations were determined on an SDS polyacrylamide gel using a BSA standard. To determine Michaëlis-Menten kinetics the following reaction mixture was prepared: 50 mM Tris, pH 7.4, 10 mM MgCl_2_, 50 ng PKD and 100 µM ATP added with 2 µCi [γ-32P]ATP (Perkin-Elmer, Massachusetts, USA). Reactions were started with Syntide-2 (Syn-2) peptide at different concentrations (15–480 µM). After 10′ (in the linear range) the reaction was stopped by spotting 30 µl on a Whatman P81 filter paper. The filter papers were washed 3 times in 0.5% phosphoric acid, followed by one wash in 100% acetone. Subsequently, the papers were air-dried and counted using the Tri-Carb 2810 TR scintillation counter (Perkin-Elmer). Non-linear regression analysis and determination of kinetic parameters was performed using Graphpad (PRISM).

### NF-κB reporter gene assays

HeLa cells per well of a 6 well plate were transiently co-transfected with 3 µg NF-κB-luciferase reporter (NF-κB-LUC), 1 µg pCS2-(n) β-Galactosidase reporter and the 1 µg cDNA of interest, using Superfect (Qiagen). 4 hours after transfection, cells were stimulated with 500 µM H_2_O_2_ for 18 hours. At the endpoint, cells were washed twice with ice-cold PBS, scraped in 250 µl Passive Lysis Buffer (Promega) and centrifuged (13,000 rpm, 10 min, 4 °C). Assays for luciferase and β-gal activities were performed using standard assays (as described in ref. 50) and measured using a Veritas luminometer (Symantec, Cupertino, CA). Luciferase activity of the NF-κB-LUC reporter construct was normalized to β-Gal activity.

### Peptide arrays

Peptide array analysis to determine PKD2 phosphorylation site specificity was carried out essentially as described^76^. Briefly, 180 peptide mixtures (50 μM) with the general sequence Y-A-x-x-x-x-x-S/T-x-x-x-x-A-G-K-K(biotin), where “x” indicates a mixture of the 17 proteogenic amino acids (excluding Cys, Ser and Thr) and S/T is an even mixture of Ser and Thr, were arrayed in 1536 well plates in 2.5 μL of 50 mM Tris, pH 7.5, 100 mM NaCl, 10 mM MgCl_2_, 1 mM DTT, 0.1% Tween 20. In each well, one “x” position was fixed as one of the 20 amino acids. In addition, two wells contained peptides in which the central S/T position was fixed as either Ser or Thr. Reactions were initiated by adding FLAG-PKD2 that had been isolated from cells either activated with PDB (not phosphorylated at Tyr-717) or H_2_O_2_ (to induce Tyr-717 phosphorylation) together with ATP (to a final concentration of 50 μM including 33 μCi/mL [γ-^33^P]ATP). Plates were incubated for 2 h at 30 C, and then 200 nl aliquots were spotted onto streptavidin coated membrane (Promega SAM^2^ biotin capture membrane). The membrane was washed extensively as described and exposed to a phosphor storage screen. Radiolabel incorporation was quantified by phosphor imaging. Data were normalized so that the average value at each peptide position was equal to 1. Data from 3 independent experiments were averaged, and log_2_ transformed data were converted to a heat map in Microsoft Excel.

## Electronic supplementary material

Supplementary information for differential regulation of PKD isoforms in oxidative stress conditions through phosphorylation of a conserved Tyr in the P + 1 loop
