# Supplementary material for: Differential regulation of PKD isoforms in oxidative stress conditions through phosphorylation of a conserved Tyr in the P+1 loop
Source: Sci Rep. 2017 Apr 20;7:887. doi: 10.1038/s41598-017-00800-w (PMC5430542; doi:10.1038/s41598-017-00800-w)

Supplementary information for:

Differential regulation of PKD isoforms in oxidative stress conditions through phosphorylation of a conserved Tyr in the P+1 loop

Mathias Cobbaut, Rita Derua, Heike Döppler, Hua Jane Lou, Sandy Vandoninck, Peter Storz, Benjamin E. Turk, Thomas Seufferlein, Etienne Waelkens, Veerle Janssens, Johan Van Lint*

Supplementary Table S1.

List of identified PTMs on PKD2 with >5 references to Mass spec/site-specific studies. Data extracted from the Phosphosite database

'site-specific' studies	proteomic studies	Modified residue	peptide sequence
0	347	S197	GARKRRLsstsLAsG
1	194	S198	ARKRRLsstsLAsGH
0	45	T199	RKRRLsstsLAsGHs
0	64	S200	KRRLsstsLAsGHsV
0	43	S203	LsstsLAsGHsVRLG
0	35	S206	tsLAsGHsVRLGtsE
0	25	T211	GHsVRLGtsEsLPCT
0	5	S212	HsVRLGtsEsLPCTA
0	91	S214	VRLGtsEsLPCTAEE
0	8	S236	LLPRRPPssssSssA
0	5	S242	PssssSssAssyTGR
0	4	Y246	sSssAssyTGRPIEL
0	13	S375	EGGKAQssLGyIPLM
0	16	Y378	KAQssLGyIPLMRVV
0	8	S387	PLMRVVQsVRHttRK
0	8	T391	VVQsVRHttRKssTt
0	5	T392	VQsVRHttRKssTtL
0	12	S395	VRHttRKssTtLREG
0	51	S396	RHttRKssTtLREGW
0	7	T412	VHYSNKDtLRKRHYW
0	12	S518	VILQDAPsAPGHAPH
5	29	S706	ARIIGEKsFRRsVVG
5	65	S710	GEKsFRRsVVGtPAy
0	15	T714	FRRsVVGtPAyLAPE
0	39	Y717	sVVGtPAyLAPEVLL
5	6	S876	QGLAERIsVL______

Supplemental Figure S2

Peptide array mediated determination of amino-acid preference of PDB stimulated (non-Tyr phosphorylated) and H_2O_2 stimulated PKD2. Quantification of three individual experiments is shown.

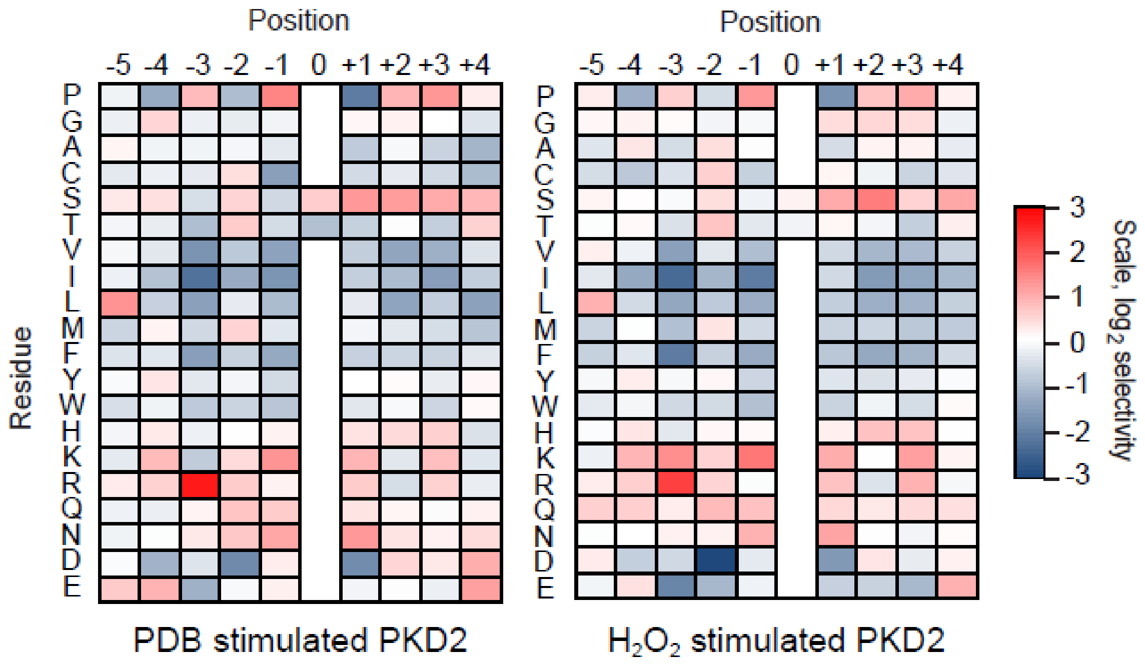

Supplementary Table S3

List of kinases that are found to be phosphorylated in the YxAPE motif. Data extracted from the Phosphosite database.

Sequence: yxAPE									
Gene	Protein	Organism	Accession	MW	Matches(yxAPE)	Start	End	Modification Sites	
AKT1	Akt1	human	UP:P31749	55686	yLAPE	315	319	Y315-p	
Akt1	Akt1	mouse	UP:P31750	55707	yLAPE	315	319	Y315-p	
Akt1	Akt1	rat	UP:P47196	55735	yLAPE	315	319	Y315-p	
AKT2	Akt2	human	UP:P31751	55769	yLAPE	316	320	Y316-p	
AKT3	Akt3	human	UP:Q9Y243	55775	yLAPE	312	316	Y312-p	
ACVRL1	ALK1	human	UP:P37023	56124	yMAPE	375	379	Y375-p	
ACVR1B	ALK4	human	UP:P36896	56807	yMAPE	380	384	Y380-p	
PRKAA1	AMPKA	human	UP:Q1313	64009	yAAPE	190	194	Y190-	

	1		1					p	
PRKAA2	AMPKA 2	human	UP:P5464 6	62320	yAAPE	179	183	Y179-p	
BMPR1 A	BMPR1 A	human	UP:P3689 4	60198	yMAPE	407	411	Y407-p	
CDK2	CDK2	human	UP:P2494 1	33930	yRAPE	168	172	Y168-p	
CHEK2	Chk2	human	UP:O9601 7	60915	yLAPE	390	394	Y390-p	
CLK1	CLK1	human	UP:P4975 9	57291	yRAPE	345	349	Y345-p	
CLK4	CLK4	human	UP:Q9HA Z1	57492	yRAPE	343	347	Y343-p	
Cit	CRIK	mouse	UP:P4902 5	23538 9	yMAPE	262	266	Y262-p	
Dyrk2	DYRK2	rat	UP:F1LYY 7	59654	yRAPE	314	318	Y314-p	
MAPK3	ERK1	human	UP:P2736 1	43136	yrAPE	210	214	Y210-p, R211-m1	
Mapk3	ERK1	mouse	UP:Q6384 4	43066	yRAPE	211	215	Y211-p	
MAPK1	ERK2	human	UP:P2848 2	41390	yrAPE	193	197	Y193-p, R194-m1	
ADRBK1	GRK2	human	UP:P2509 8	79574	yMAPE	356	360	Y356-p	
Adrbk1	GRK2	mouse	UP:Q99M K8	79639	yMAPE	356	360	Y356-p	
ADRBK2	GRK3	human	UP:P3562 6	79710	yMAPE	356	360	Y356-p	
Adrbk2	GRK3	mouse	UP:Q3UY H7	79657	yMAPE	356	360	Y356-p	
GSK3A	GSK3A	human	UP:P4984 0	50981	yRAPE	285	289	Y285-p	
GSK3B	GSK3B	human	UP:P4984 1	46744	yRAPE	222	226	Y222-p	
IKBKB	IKKB	human	UP:O1492 0	86564	yLAPE	188	192	Y188-p	
Ikbkb	IKKB	mouse	UP:O8835 1	86690	yLAPE	188	192	Y188-p	
UHMK1	KIS	human	UP:Q8TAS 1	46546	yRAPE	197	201	Y197-p	
LRRK1	LRRK1	human	UP:Q38S D2	22539 3	yQAPE	1430	1434	Y1430 -p	
Lrrk1	LRRK1	mouse	UP:Q3UH C2	22547 0	yQAPE	1430	1434	Y1430 -p	
MAPKA PK3	MAPKA PK3	human	UP:Q1664 4	42987	yVAPE	208	212	Y208-p	
Mark1	MARK1	mouse	UP:Q8VHJ 5	88335	yAAPE	222	226	Y222-p	
Mark2	MARK2	mouse	UP:Q0551 2	86306	yAAPE	215	219	Y215-p	

Mark3	MARK3	mouse	UP:Q03141	84390	yAAPE	218	222	Y218-p	
Mark4	MARK4	mouse	UP:Q8CIP4	82644	yAAPE	221	225	Y221-p	
Map3k3	MEKK3	mouse	UP:Q61084	70776	yQAPE	155	159	Y155-p	
MAP2K3	MKK3	human	UP:P46734	39318	yMAPE	230	234	Y230-p	
MAP2K6	MKK6	human	UP:P52564	37492	yMAPE	219	223	Y219-p	
MAP2K7	MKK7	human	UP:O14733	47485	yMAPE	283	287	Y283-p	
STK16	MPSK1	human	UP:O75716	34656	yRAPE	198	202	Y198-p	
STK38	NDR1	human	UP:Q15208	54190	yIAPE	288	292	Y288-p	
Stk38	NDR1	mouse	UP:Q91VJ4	54174	yIAPE	288	292	Y288-p	
STK38L	NDR2	human	UP:Q9Y2H1	54003	yIAPE	289	293	Y289-p	
NR1D1	NR1D1	human	UP:P20393	66805	yAAPE	394	398	Y394-p	
RPS6KA1	p90RSK	human	UP:Q15418	82723	yMAPE	228	232	Y228-p	
Rps6ka1	p90RSK	mouse	UP:P18653	81595	yMAPE	228	232	Y228-p	
PRKCA	PKCA	human	UP:P17252	76750	yIAPE	504	508	Y504-p	
Prkca	PKCA	mouse	UP:P20444	76852	yIAPE	504	508	Y504-p	
Prkca	PKCA	rat	UP:P05696	76792	yIAPE	504	508	Y504-p	
PRKCB	PKCB	human	UP:P05771	76869	yIAPE	507	511	Y507-p	
Prkcb	PKCB	mouse	GP:EDL17280	73235	yIAPE	474	478	Y474-p	
Prkcb	PKCB	rat	UP:P04410	76751	yIAPE	507	511	Y507-p	
Prkcb	PKCB iso2	rat	UP:P68403-2	76894	yIAPE	507	511	Y507-p	
PRKCD	PKCD	human	UP:Q05655	77505	yIAPE	514	518	Y514-p	
Prkcd	PKCD	rat	UP:P09215	77520	yIAPE	512	516	Y512-p	
PRKCE	PKCE	human	UP:Q02156	83674	yIAPE	573	577	Y573-p	
PRKCG	PKCG	human	UP:P05129	78448	yIAPE	521	525	Y521-p	
Prkcg	PKCG	mouse	UP:P63318	78358	yIAPE	521	525	Y521-p	
Prkcg	PKCG	rat	UP:P6331	78358	yIAPE	521	525	Y521-	

			9					p	
PRKCQ	PKCT	human	UP:Q04759	81865	yIAPE	545	549	Y545-p	
PRKCZ	PKCZ	human	UP:Q05513	67660	yIAPE	417	421	Y417-p	
PLK1	PLK1	human	UP:P53350	68255	yIAPE	217	221	Y217-p	
PRKD1	PRKD1	human	UP:Q15139	101704	yLAPE	749	753	Y749-p	
Prkd1	PRKD1	mouse	UP:Q62101	102037	yLAPE	755	759	Y755-p	
PRKD2	PRKD2	human	UP:Q9BZL6	96750	yLAPE	717	721	Y717-p	
PRKD3	PRKD3	human	UP:O94806	100471	yLAPE	742	746	Y742-p	
Prkd3	PRKD3	mouse	UP:Q8K1Y2	100078	yLAPE	741	745	Y741-p	
PRPF4B	PRP4	human	UP:Q13523	116987	yRAPE	855	859	Y855-p	
RPS6KA3	RSK2	human	UP:P51812	83736	yMAPE	234	238	Y234-p	
Rps6ka3	RSK2	mouse	UP:P18654	83694	yMAPE	234	238	Y234-p	
RPS6KA6	RSK4	human	UP:Q9UK32	83872	yMAPE	239	243	Y239-p	
Rps6ka6	RSK4	mouse	UP:Q7TPS0	86571	yMAPE	259	263	Y259-p	
TGFBR2	TGFBR2	human	UP:P37173	64568	yMAPE	424	428	Y424-p	

Supplemental Figure S4

Uncropped blots of Figure 1c

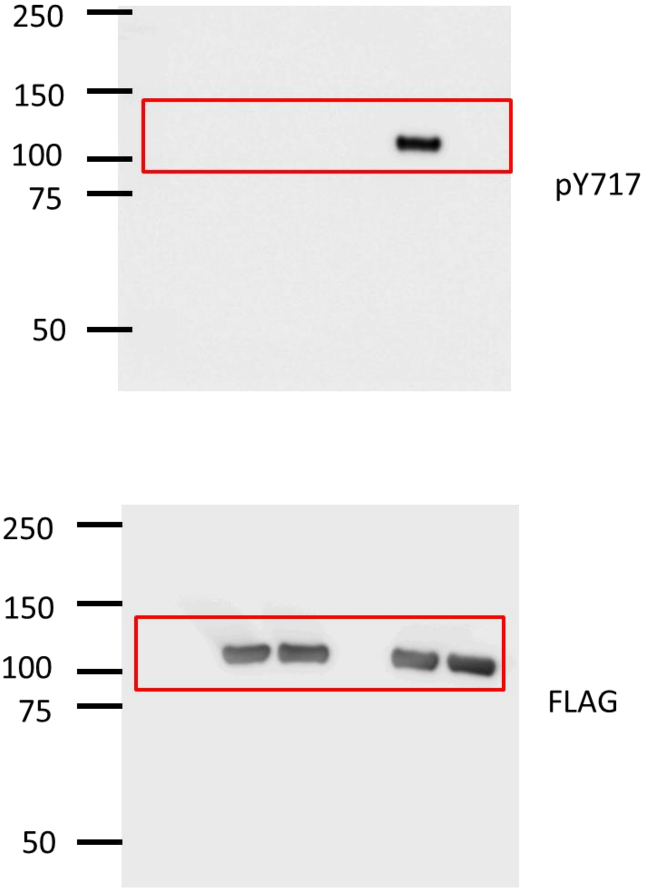

Supplemental Figure S5

Uncropped blots of Figure 1d

(2 experiments loaded)

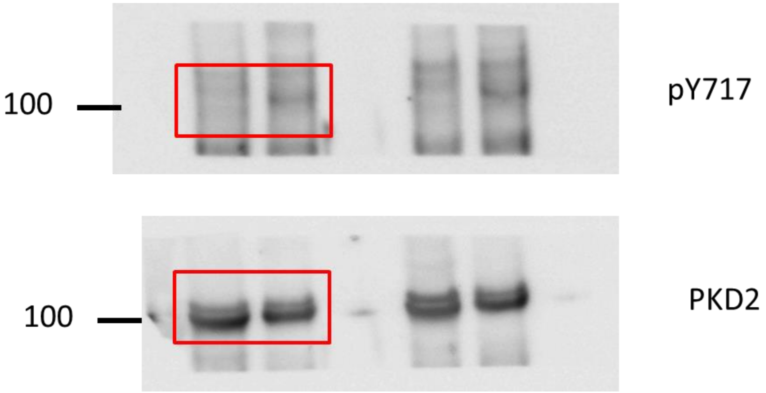

Supplemental Figure S6

Uncropped blots of Figure 1e

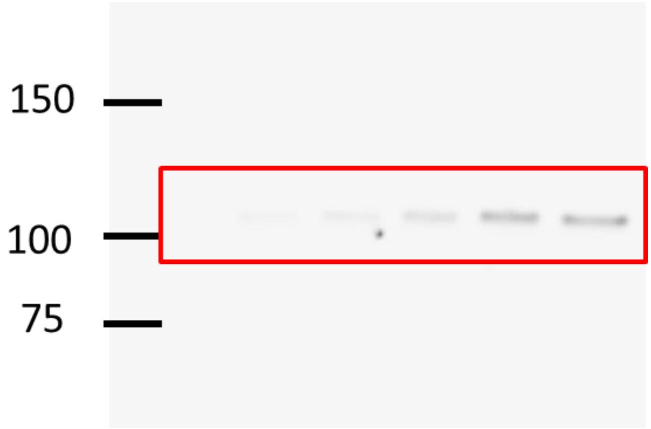

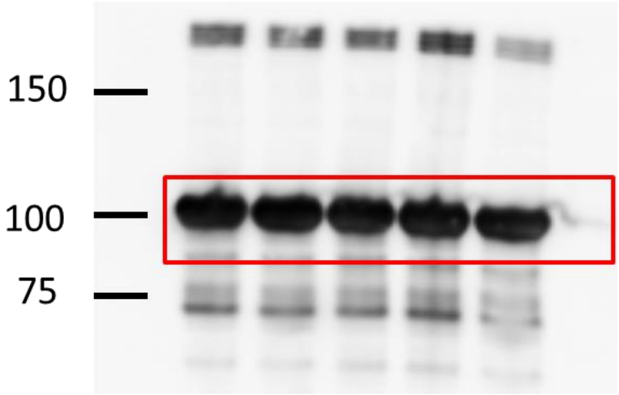

Supplemental Figure S7

Uncropped blots of Figure 1f

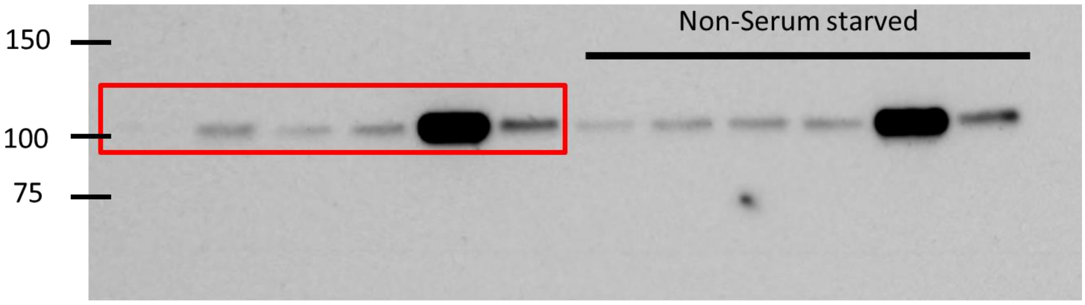

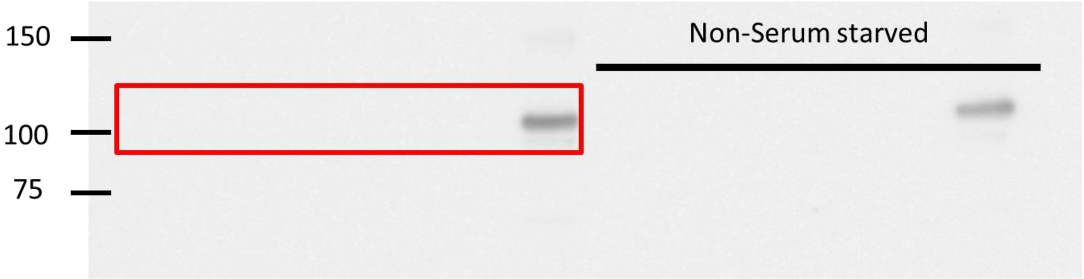

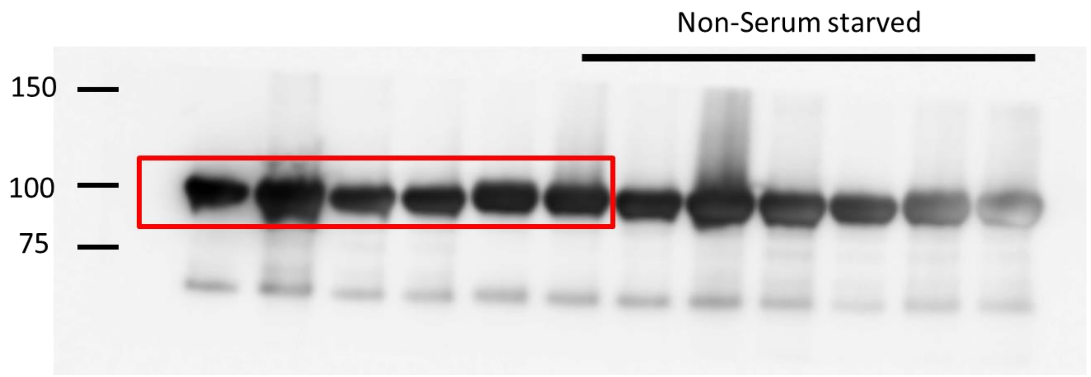

Supplemental Figure S8

Uncropped blots of Figure 2a

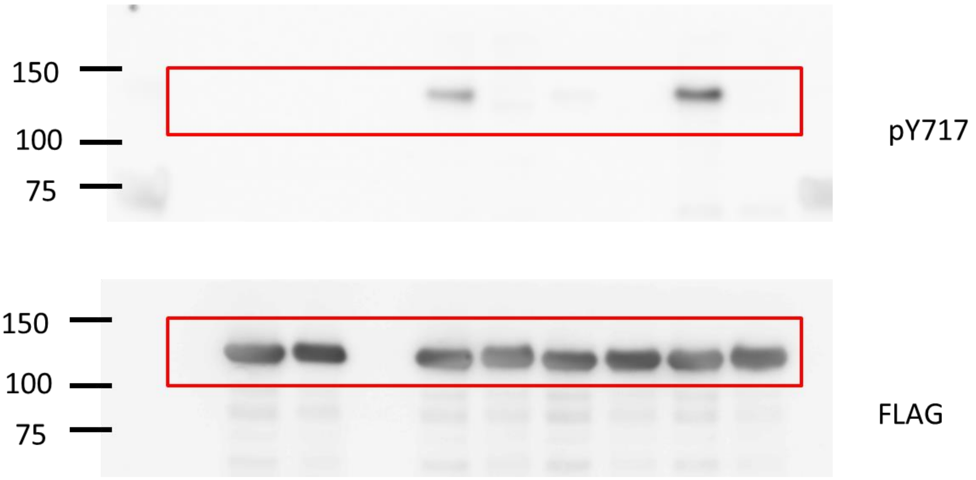

Supplemental Figure S9

Uncropped blots of Figure 2b

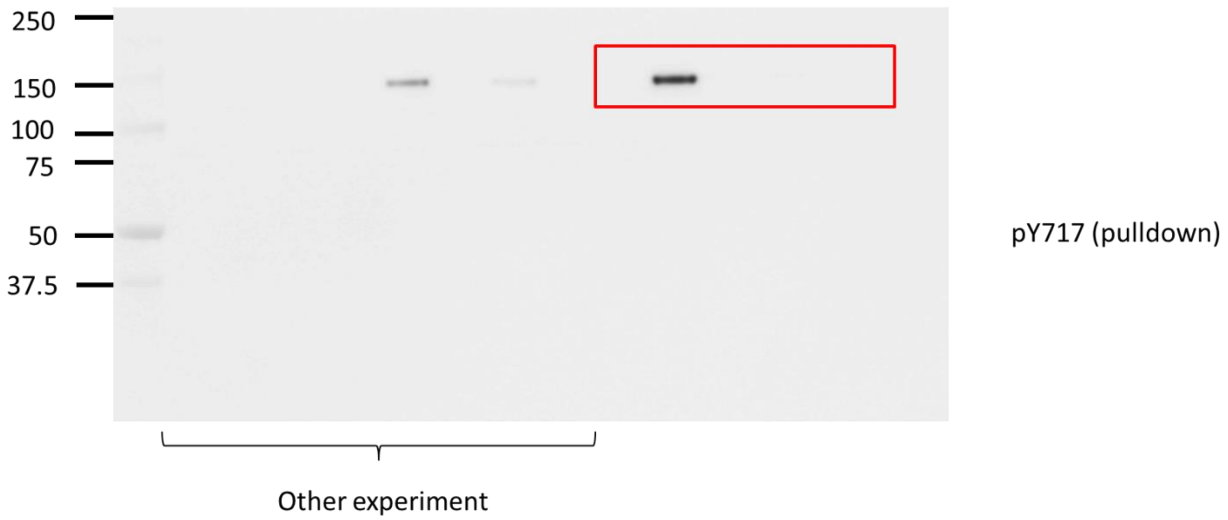

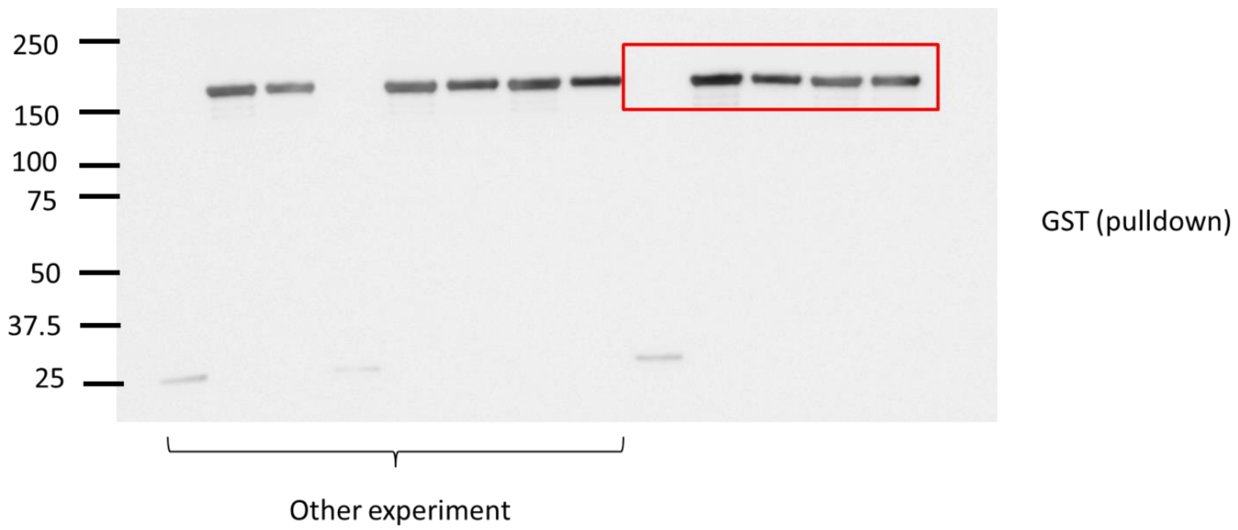
250
150
100
75
50
37.5
25
GST (pulldown)
Other experiment

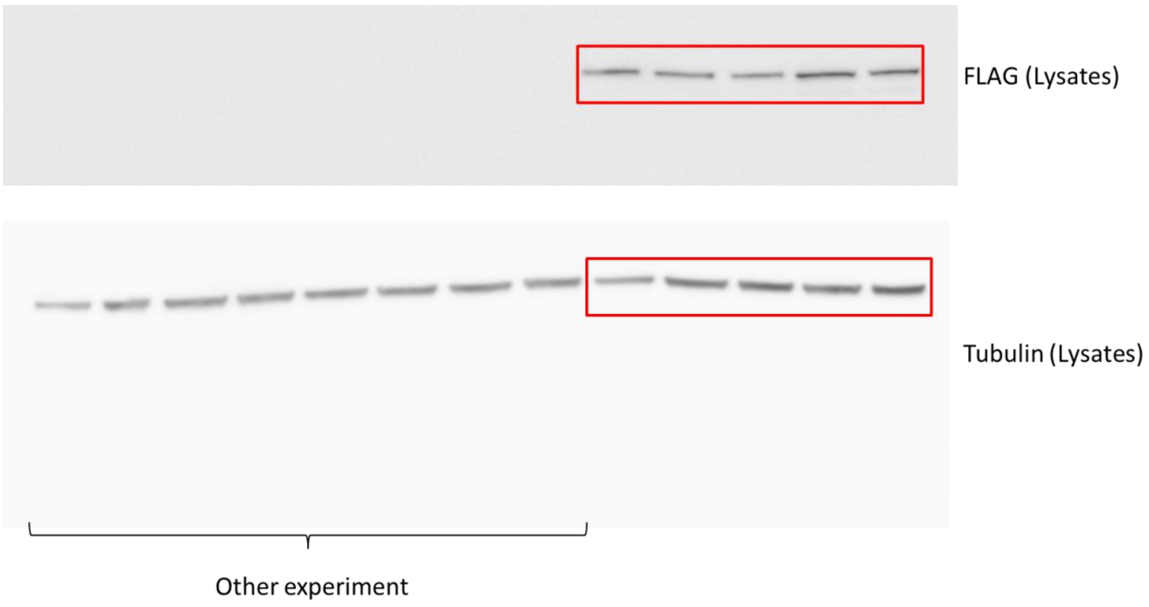
FLAG (Lysates)
Tubulin (Lysates)
Other experiment

Supplemental Figure S10

Uncropped blots of Figure 2c

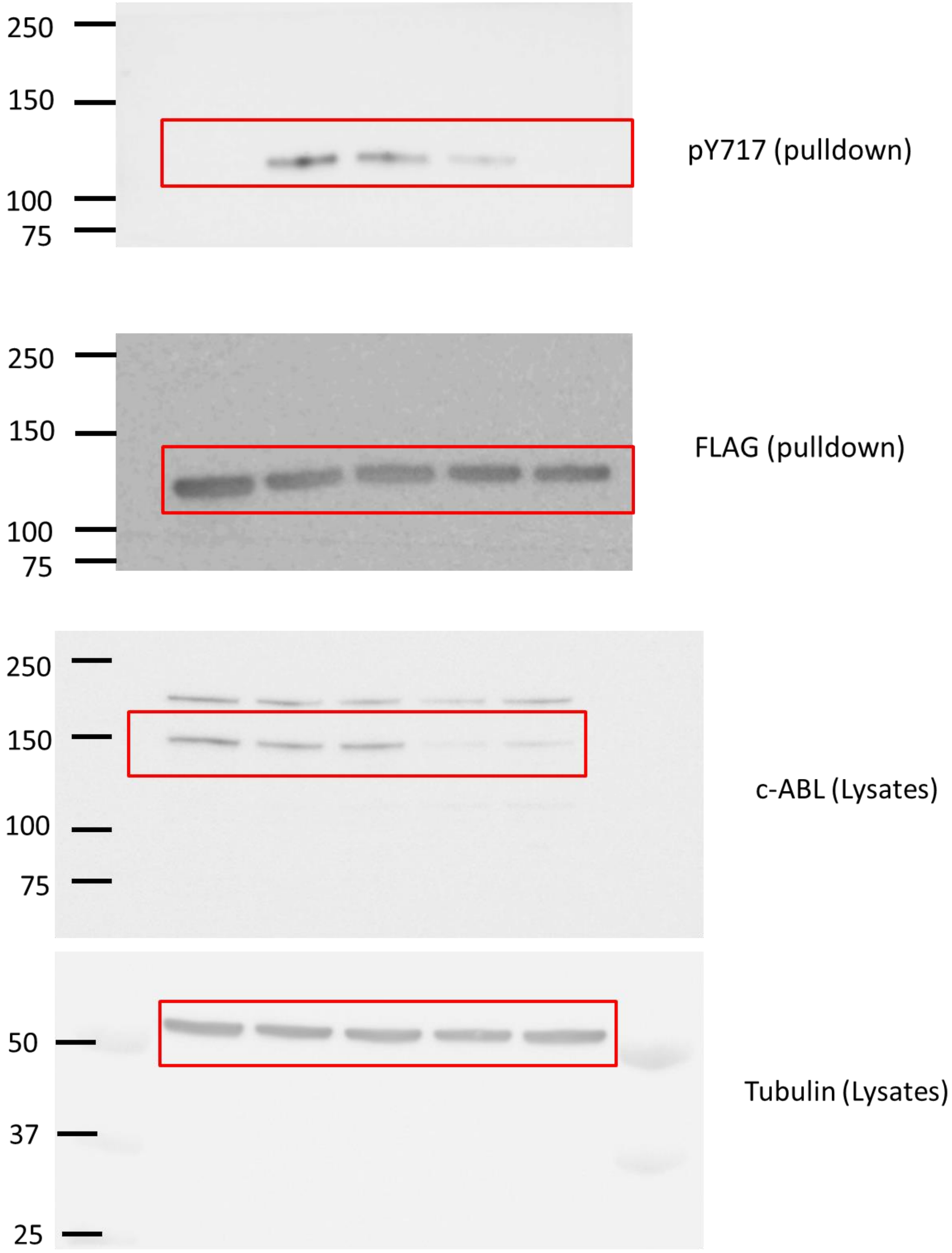

Supplemental Figure S11

Uncropped blots of Figure 2d

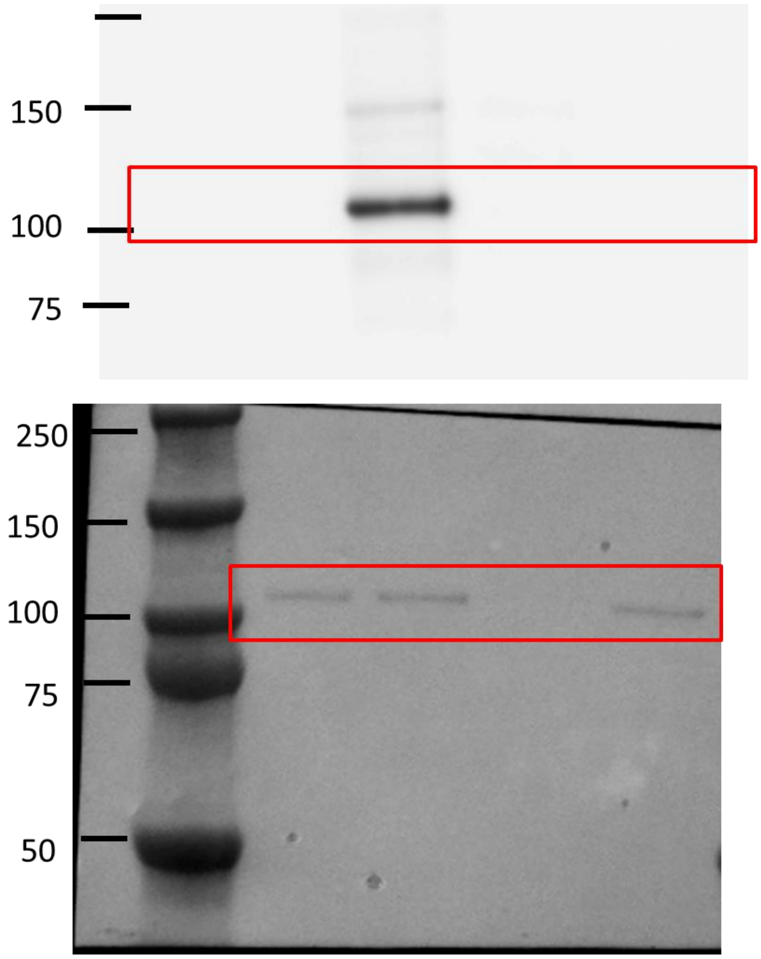

Supplemental Figure S12

Uncropped blots of Figure 3c

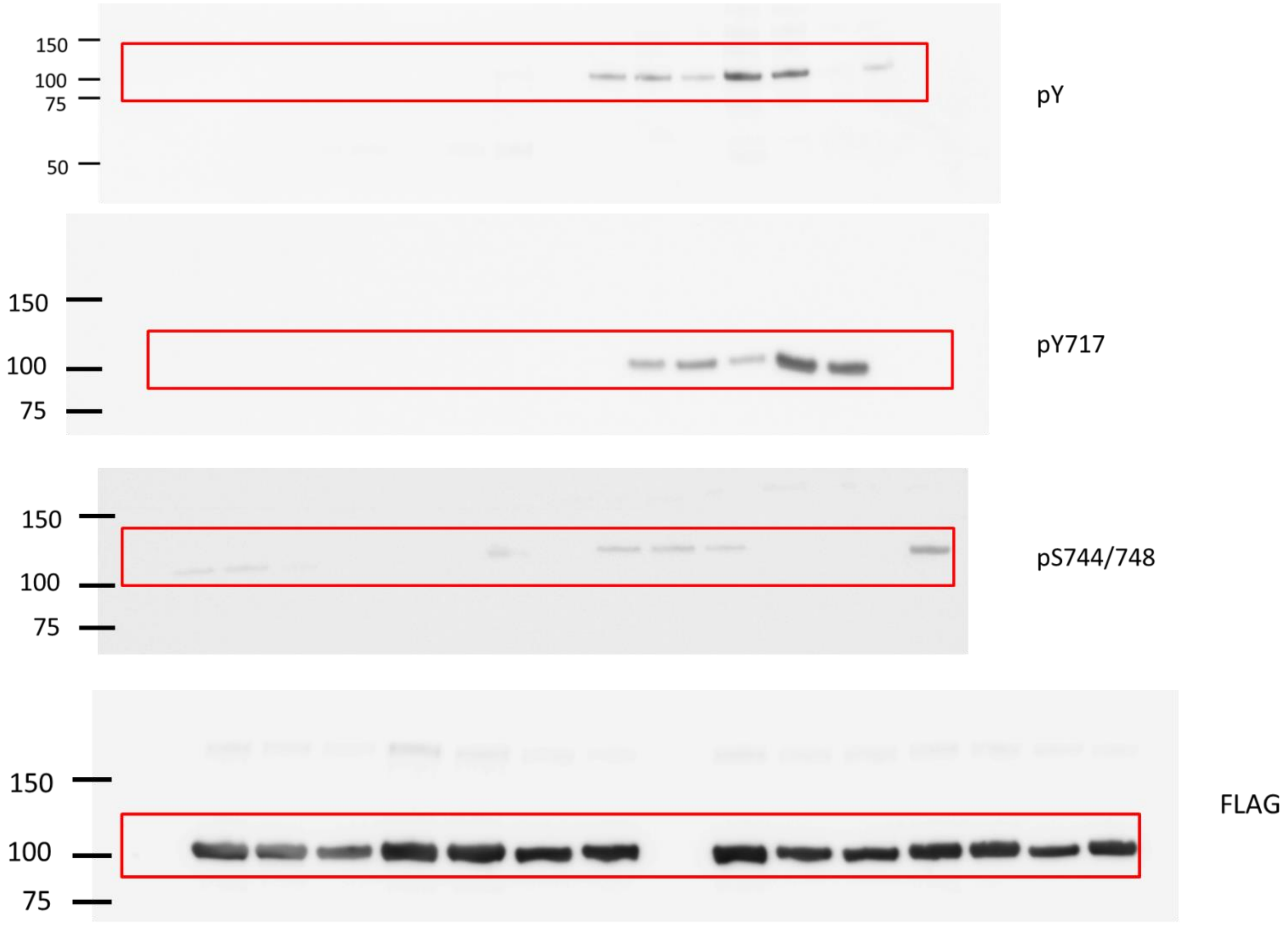

Supplemental Figure S13

Uncropped blots of Figure 3d

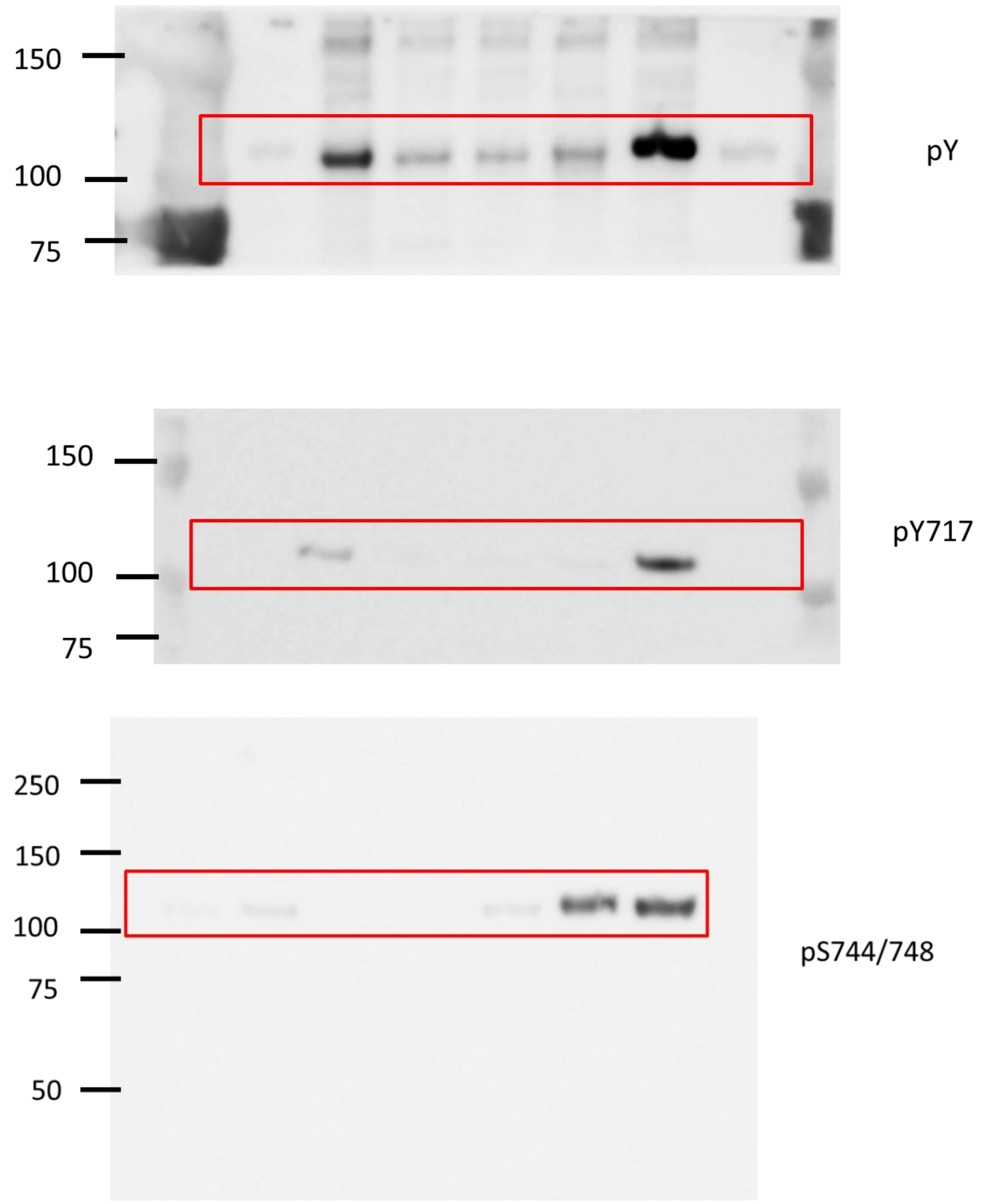

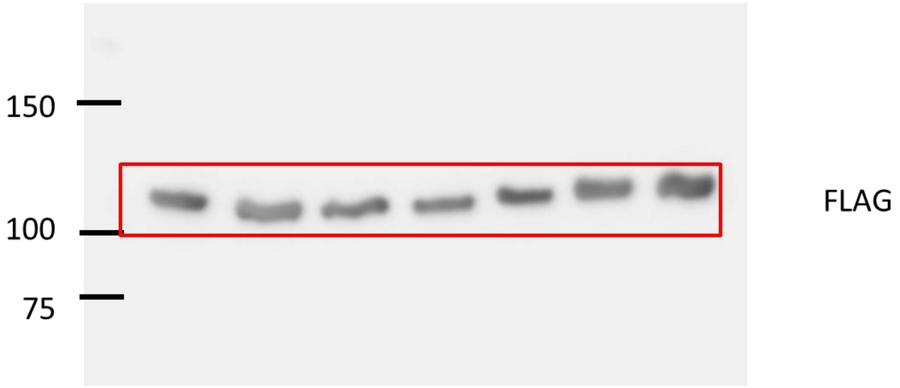

Supplemental Figure S14

Uncropped blots of Figure 3e

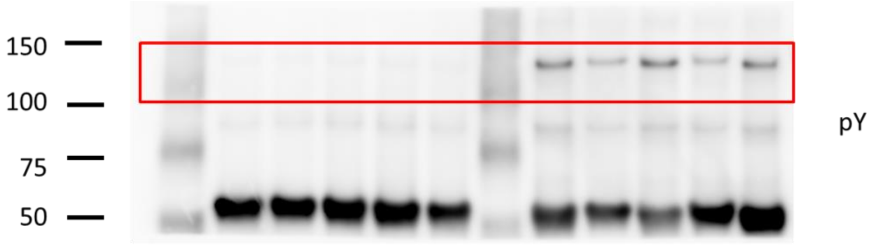

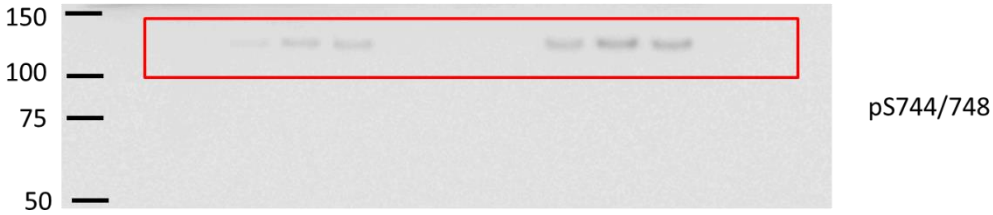

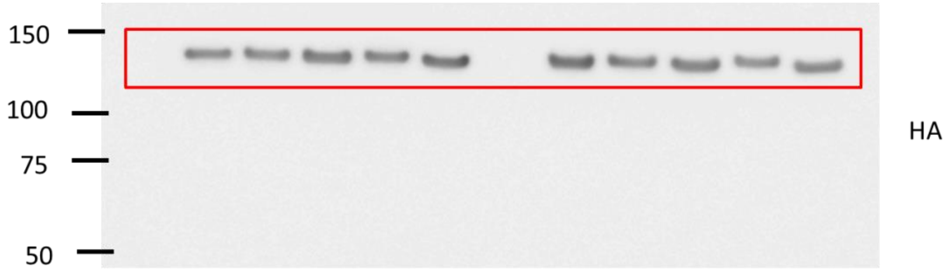

Supplemental Figure S15

Uncropped blots of Figure 4a

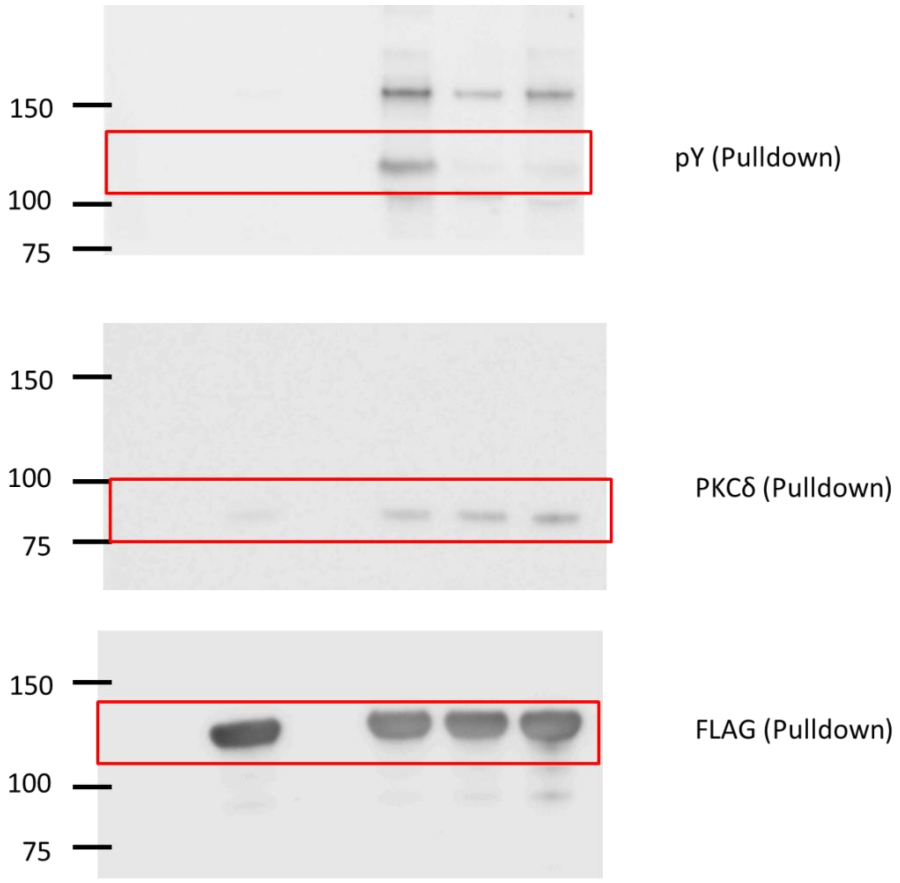

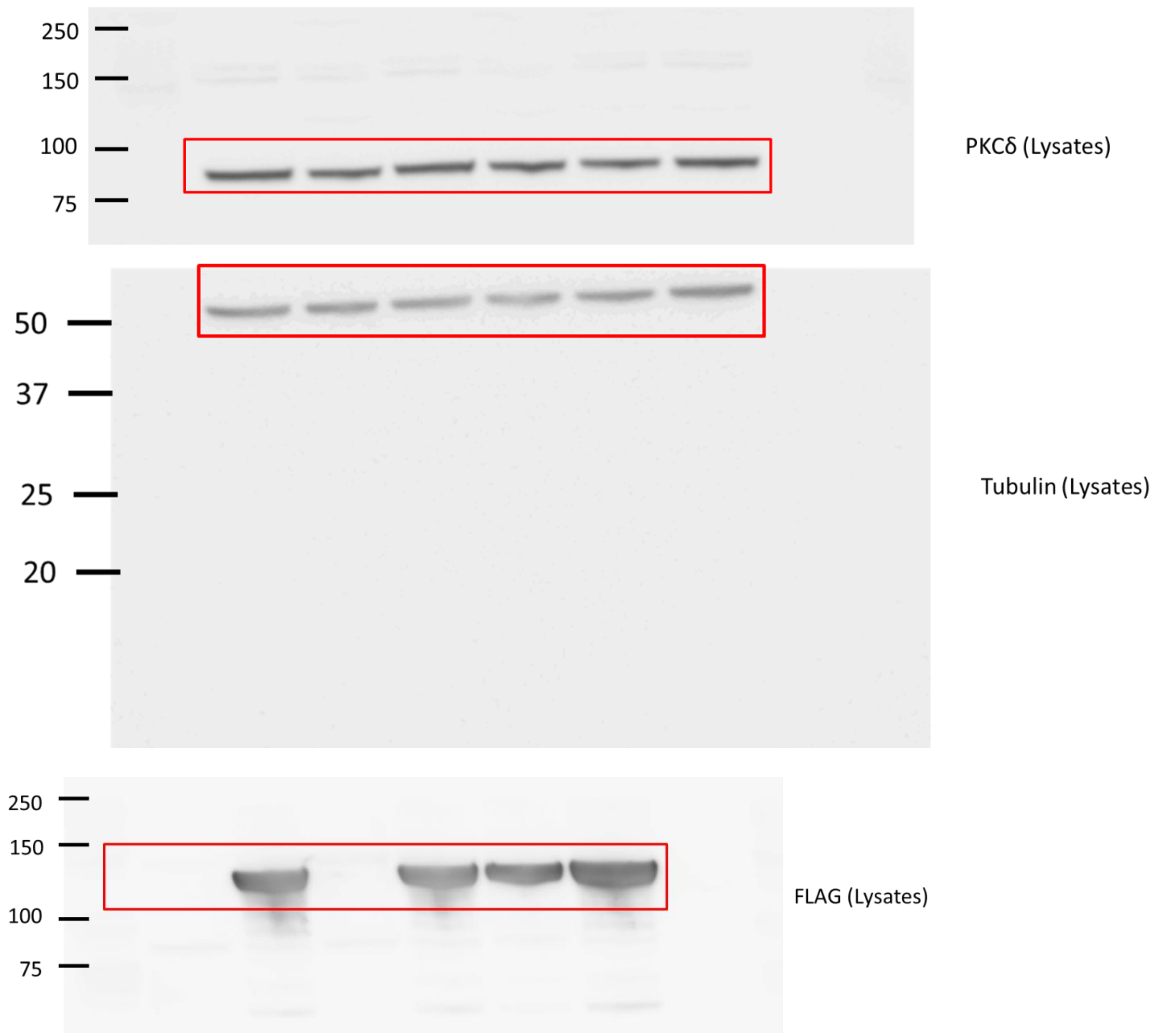
250
150
100
75
PKCδ (Lysates)
50
37
25
20
Tubulin (Lysates)
250
150
100
75
FLAG (Lysates)

Supplemental Figure S16

Uncropped blots of Figure 4b

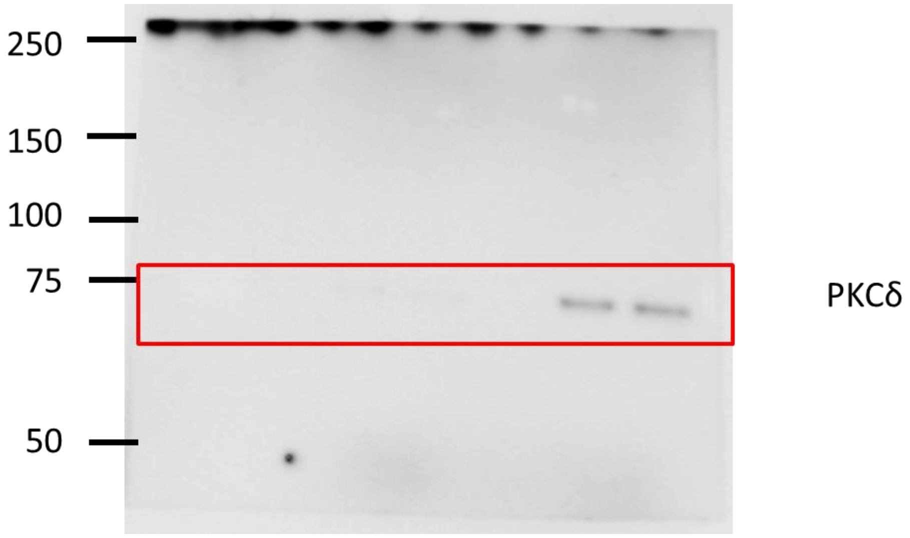

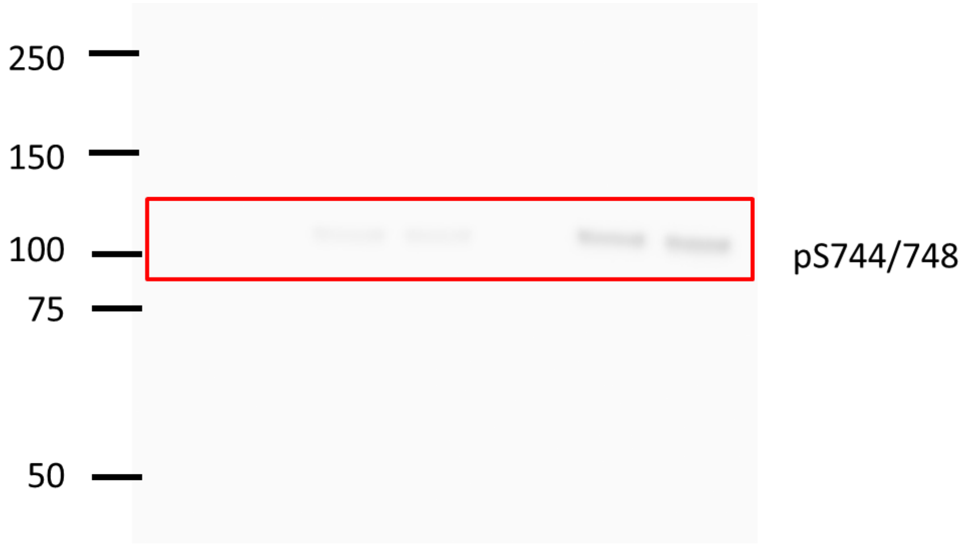

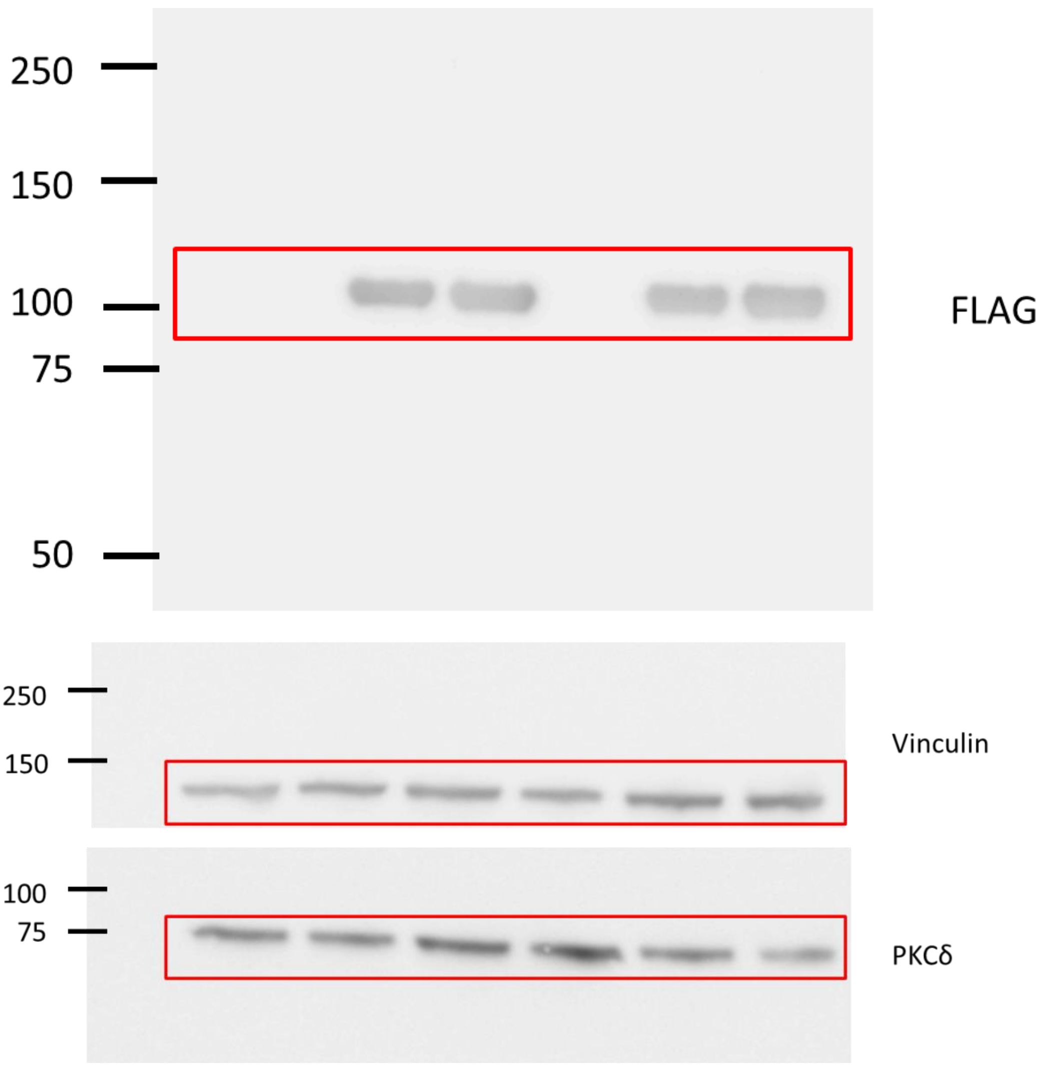
250
150
100
75
50
FLAG
250
150
Vinculin
100
75
PKCδ

Supplemental Figure S17

Uncropped blots of Figure 4d

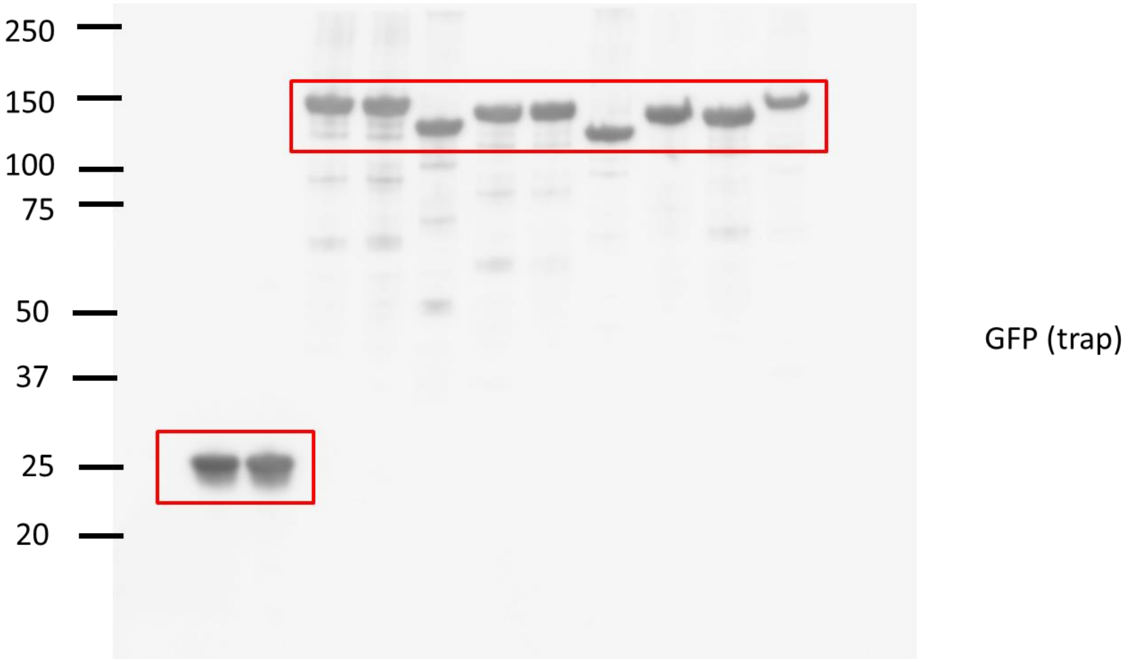

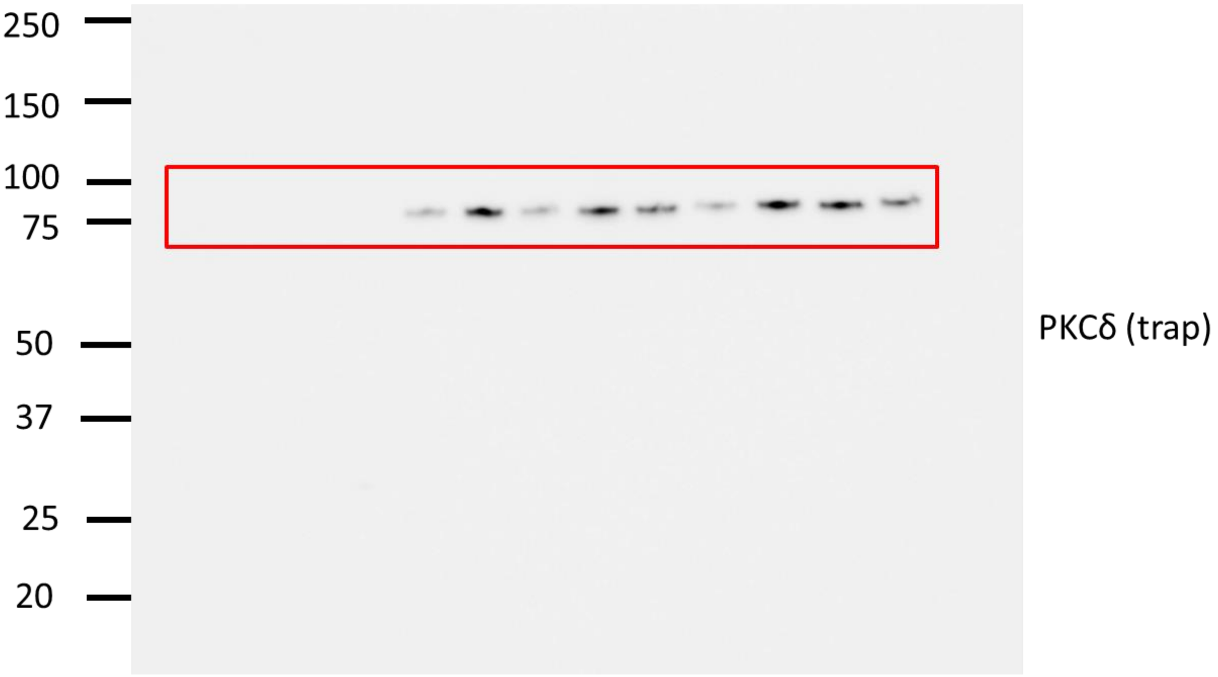

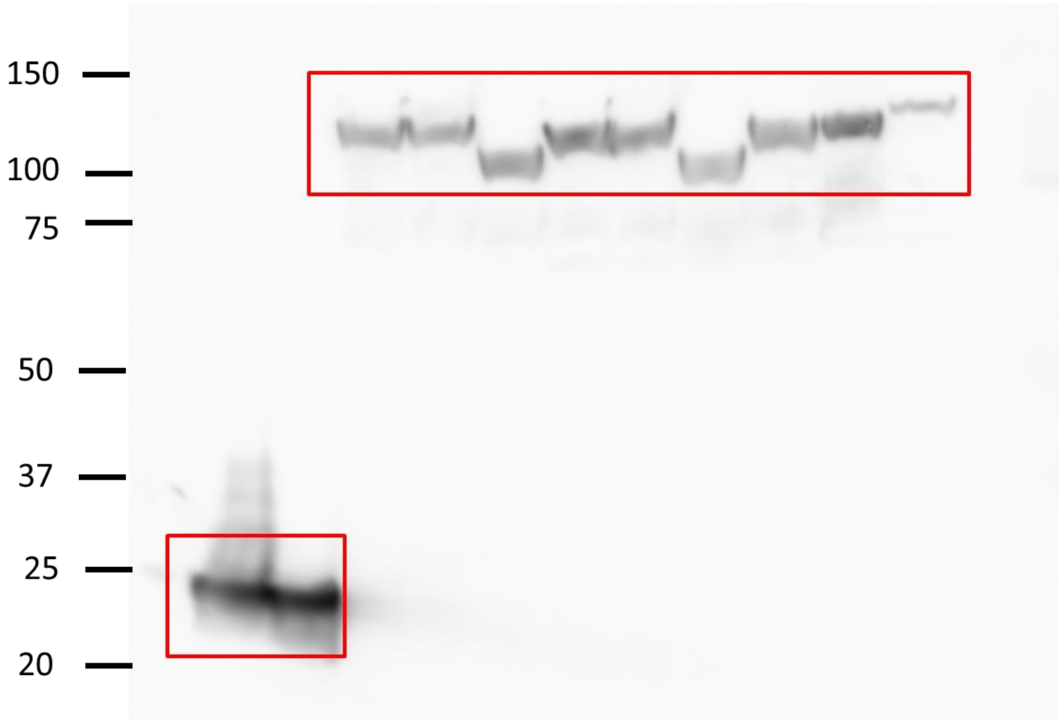

GFP (lysates)

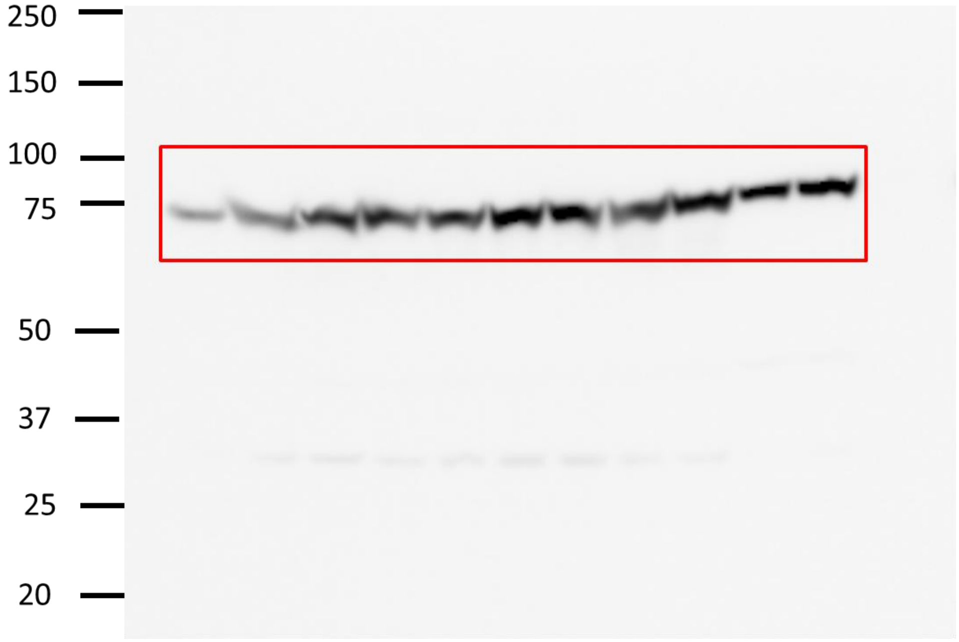

PKCδ (lysates)

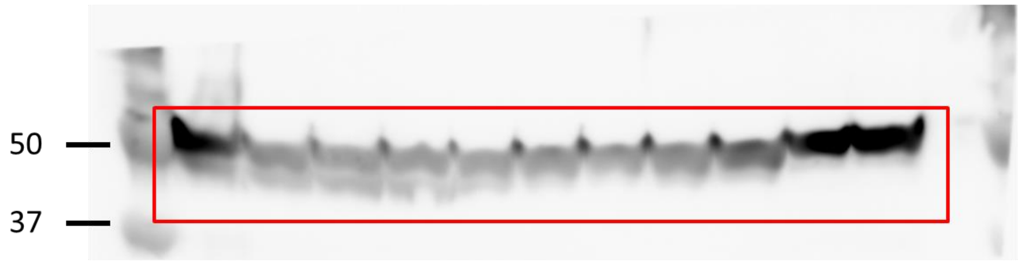

Tubulin (lysates)

Supplemental Figure S18

Uncropped blots of Figure 5a

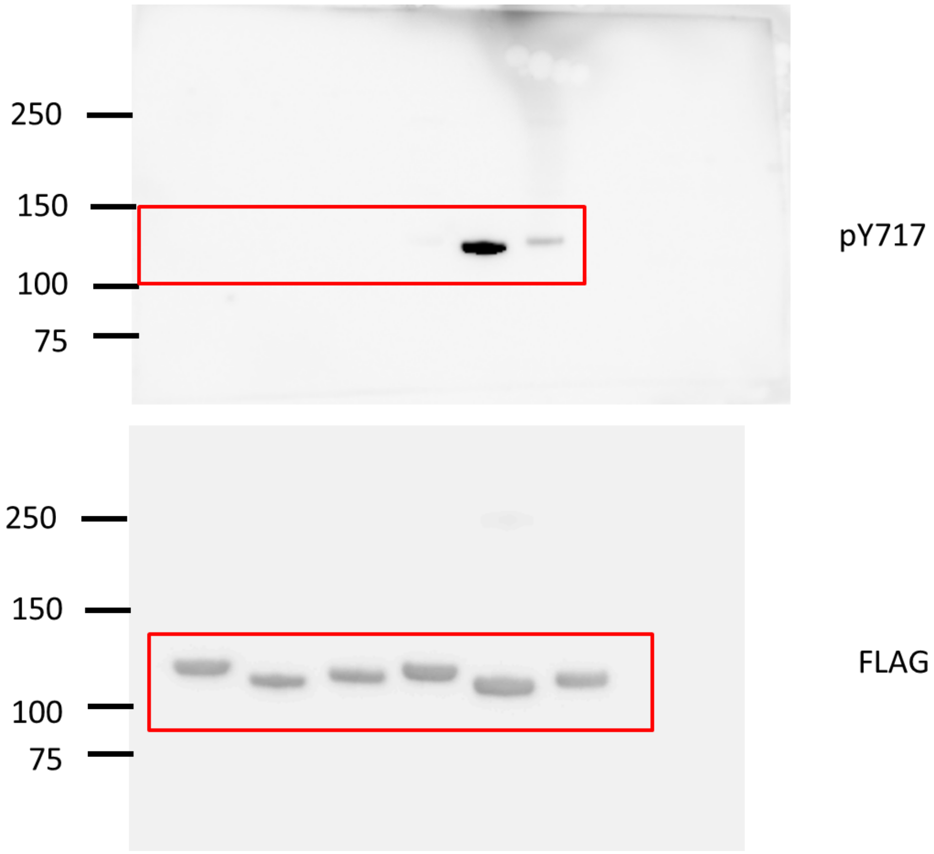

Supplemental Figure S19

Uncropped blots of Figure 5b

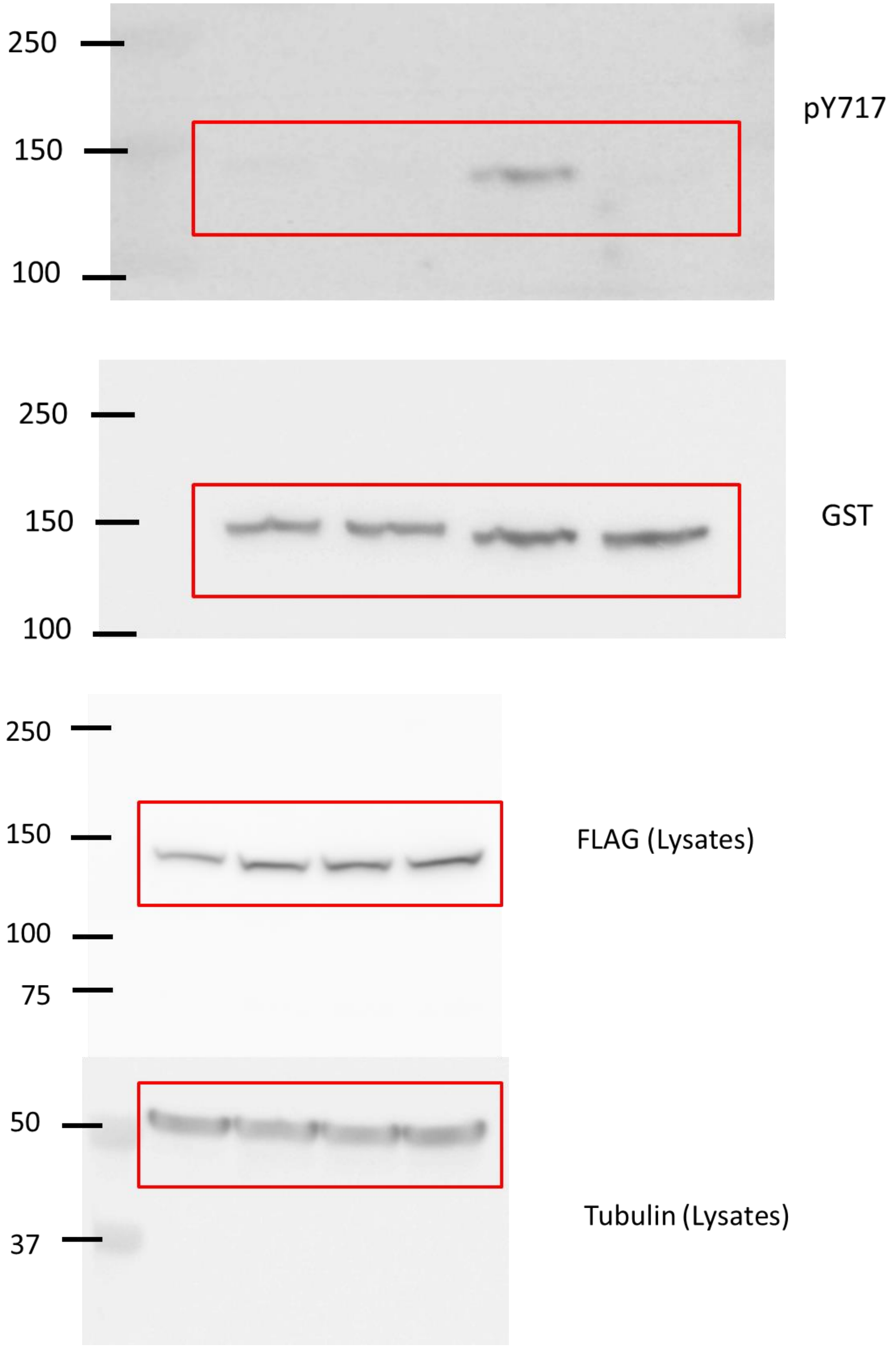

Supplemental Figure S20

Uncropped blots of Figure 5d

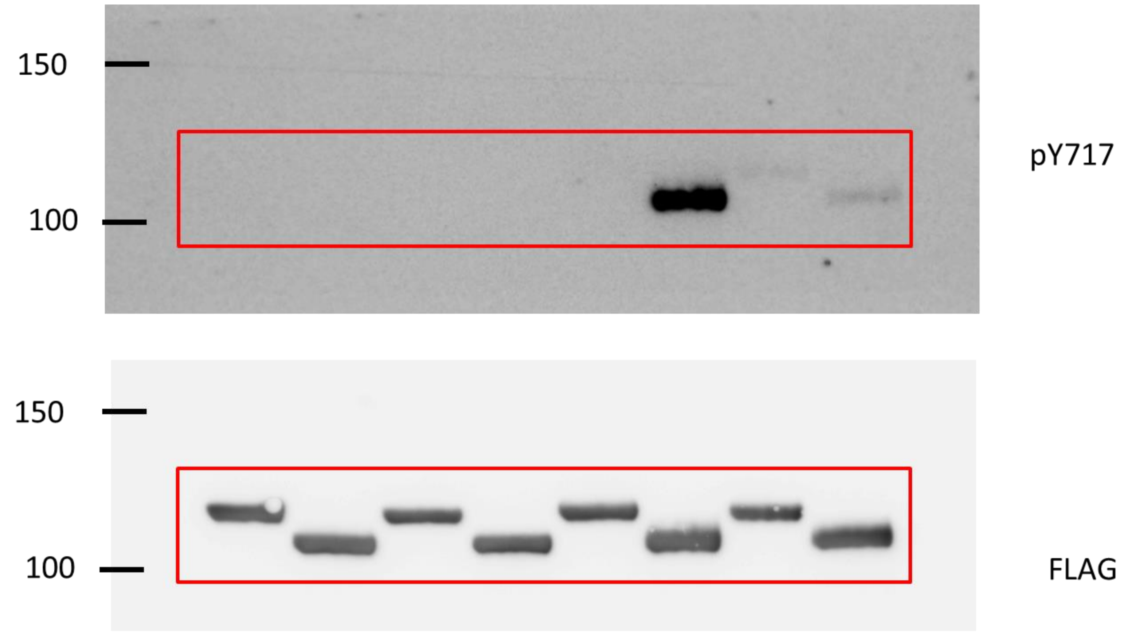

Supplemental Figure S21

Uncropped blots of Figure 5e

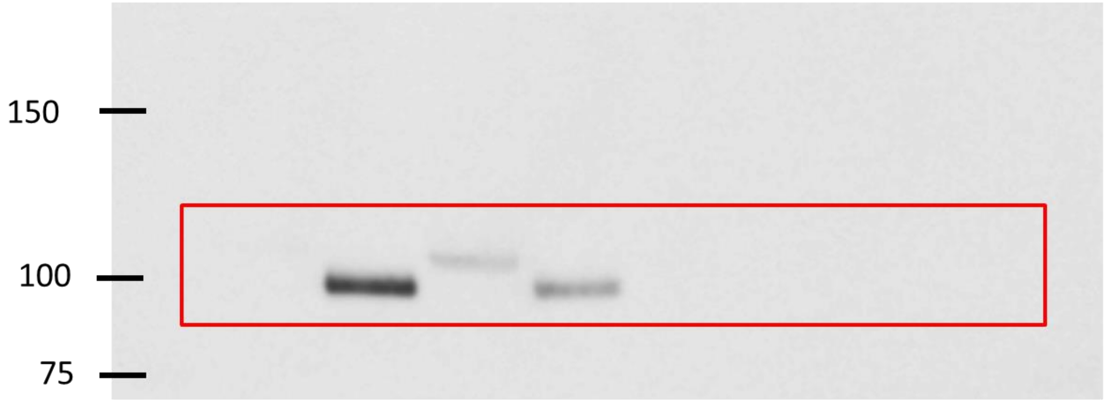

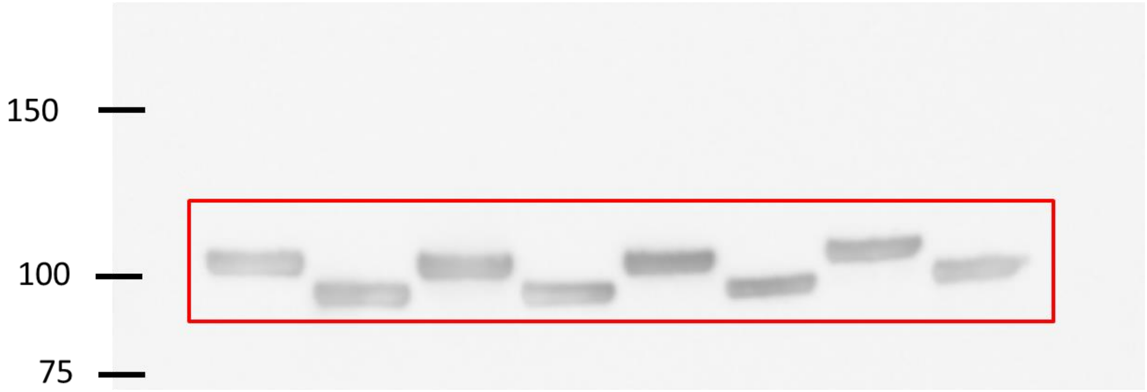

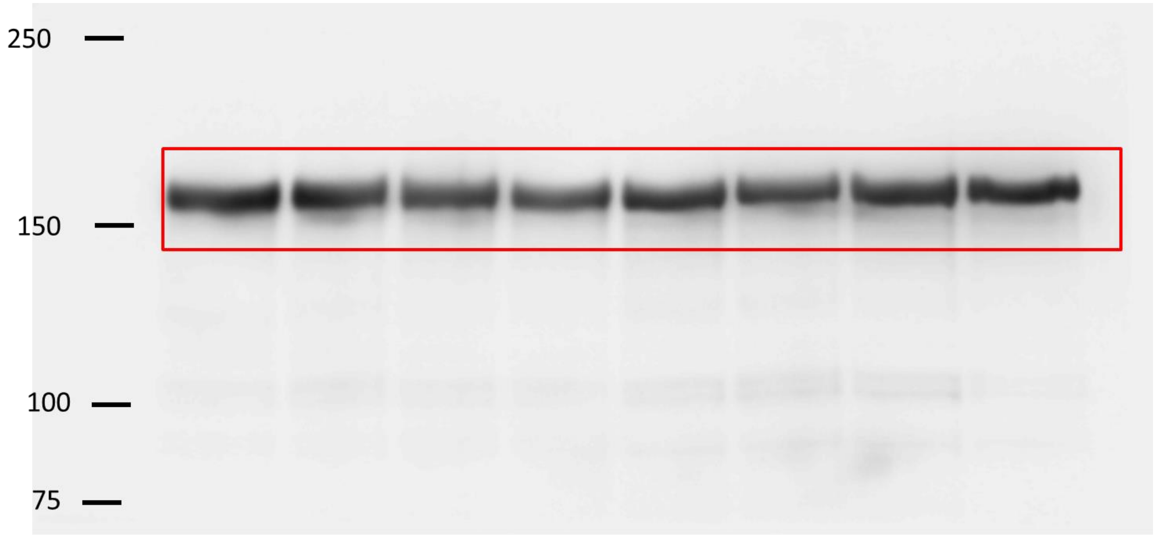
250
150
100
75

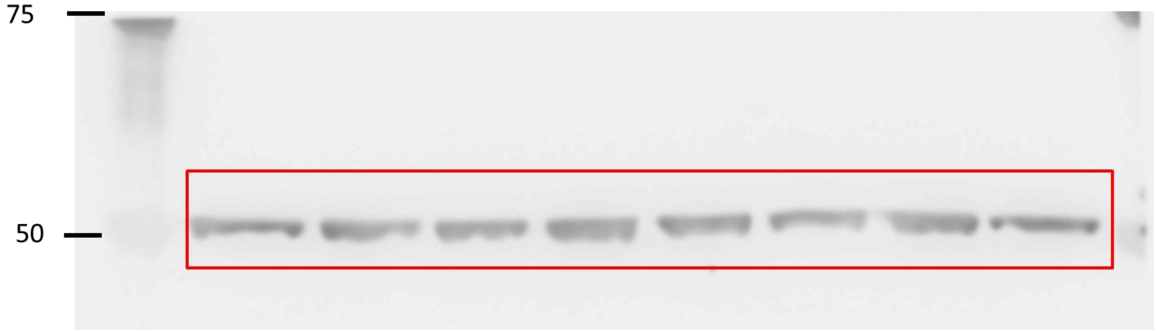
75
50

Supplemental Figure S22

Uncropped blots of Figure 6c

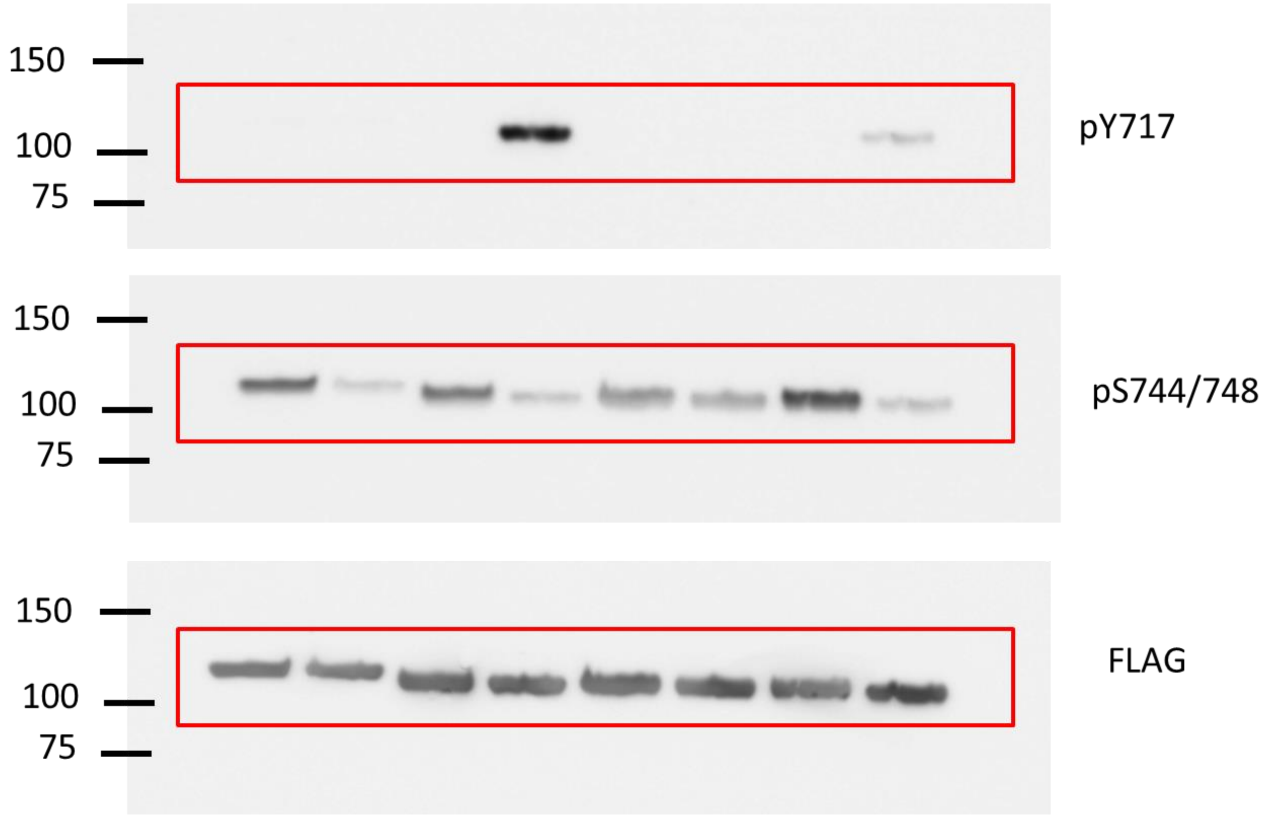

Supplemental Figure S23

Uncropped blots of Figure 6d

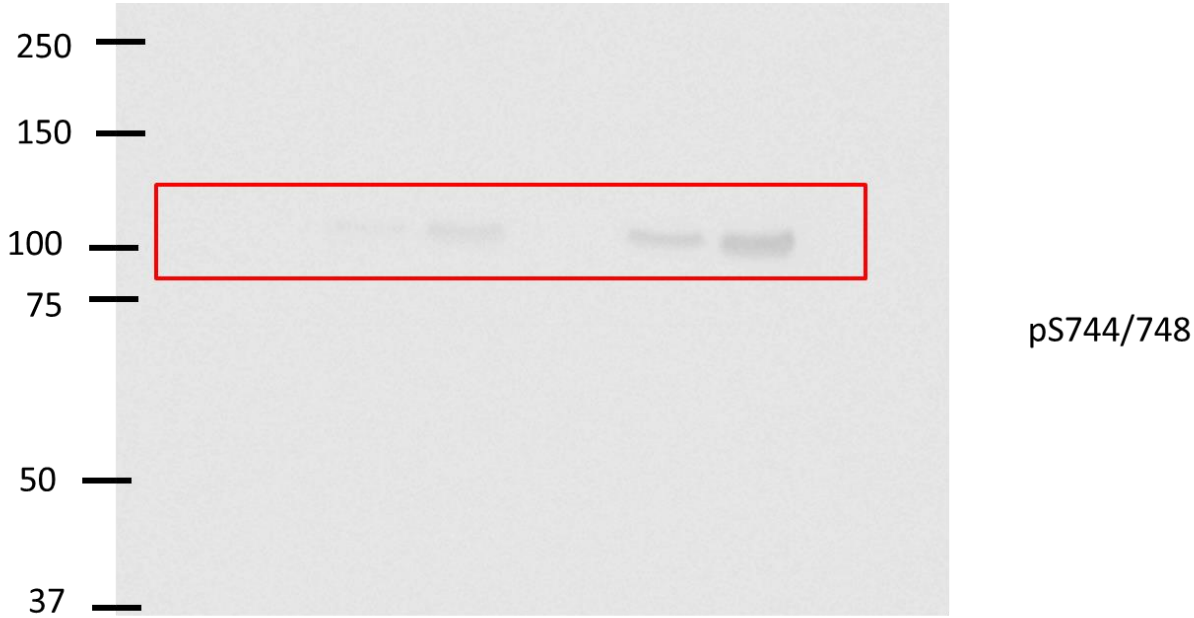

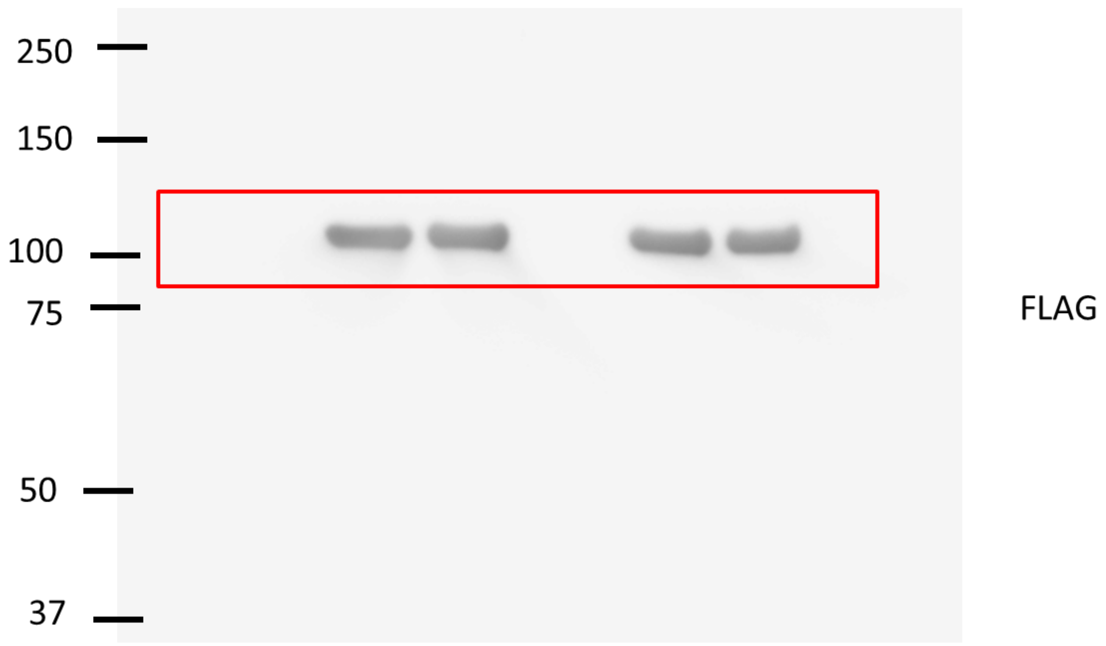
250
150
100
75
50
37
FLAG

Supplemental Figure S24

Uncropped blots of Figure 7a

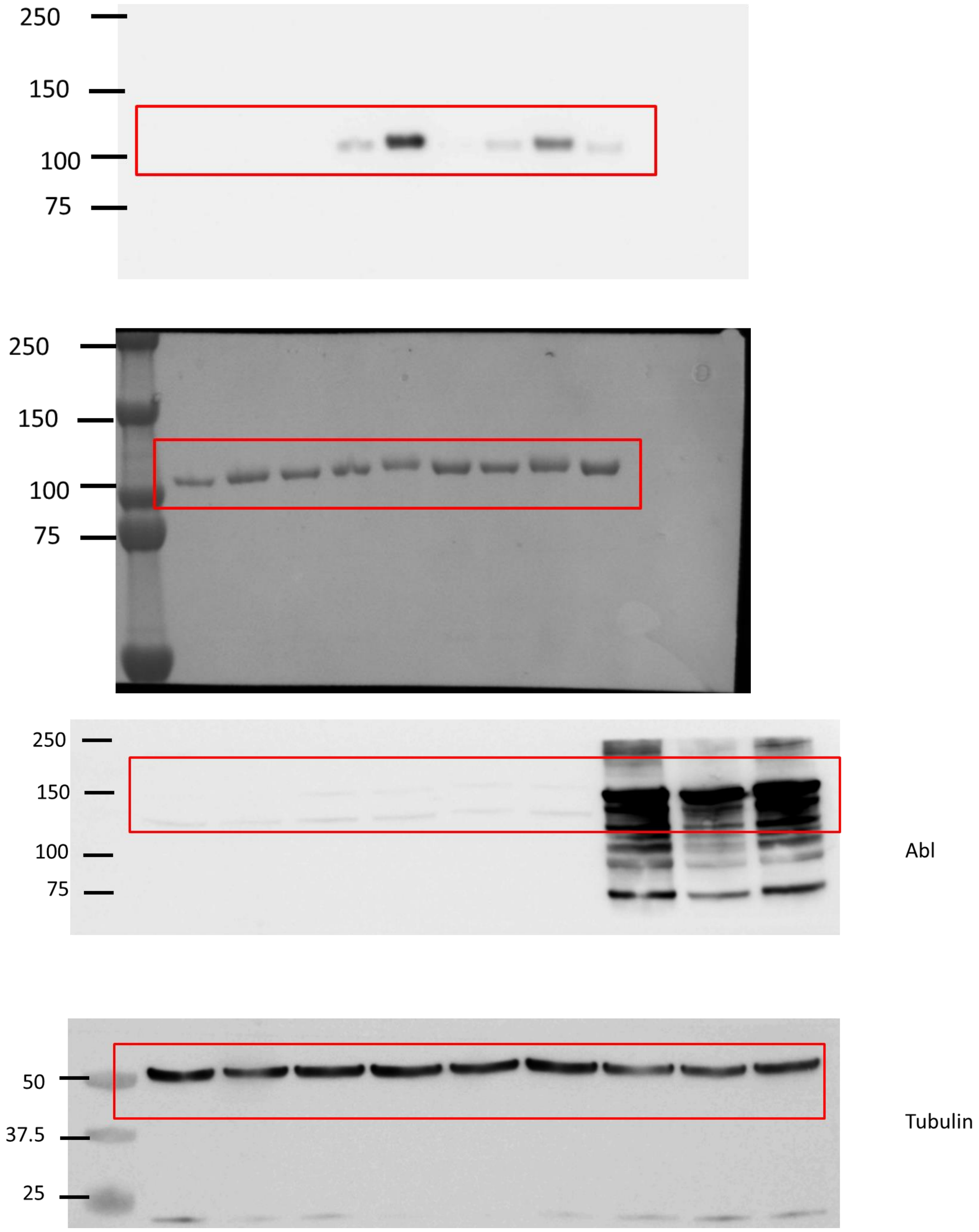

Supplemental Figure S25

Uncropped blots of Figure 7c

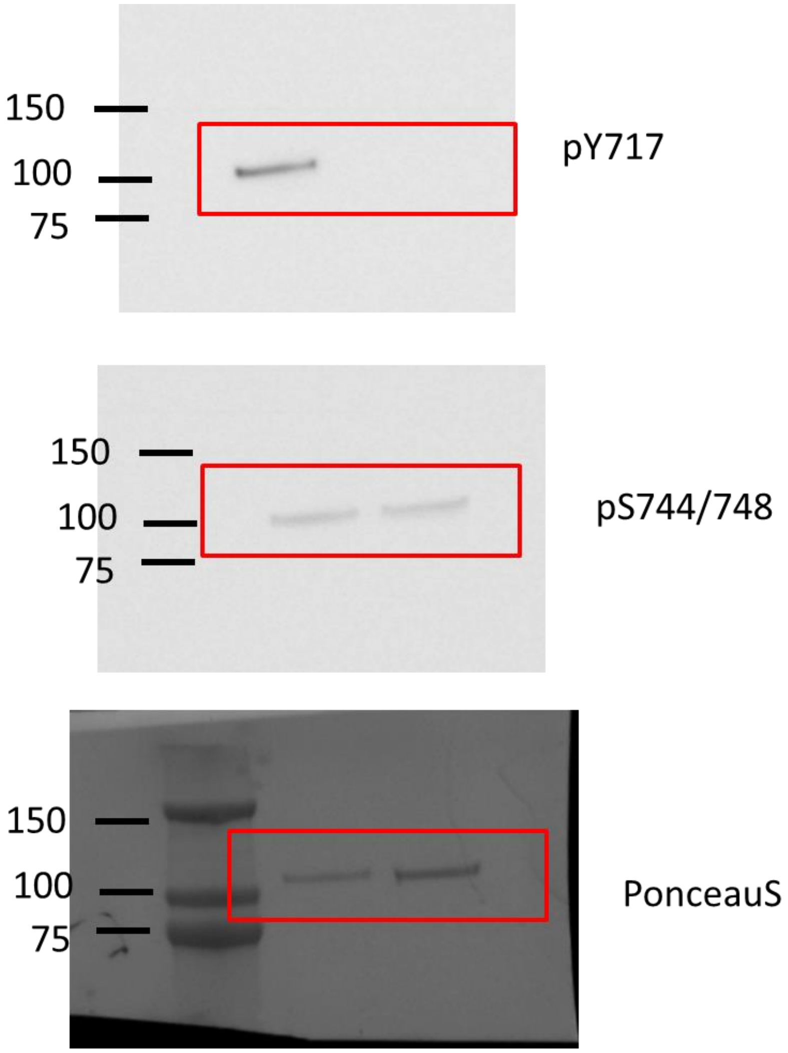

Supplement: Supplementary file 1 — Supplementary information for differential regulation of PKD isoforms in oxidative stress conditions through phosphorylation of a conserved Tyr in the P + 1 loop [file 41598_2017_800_MOESM1_ESM.pdf]
